# Synthesis and Characterization of Phosphorus- and Carborane-Containing Polyoxanorbornene Block Copolymers

**DOI:** 10.3390/polym11040613

**Published:** 2019-04-03

**Authors:** Gizem Kahraman, De-Yi Wang, Jonas von Irmer, Markus Gallei, Evamarie Hey-Hawkins, Tarik Eren

**Affiliations:** 1 Chemistry Department, Yildiz Technical University, 34220 Istanbul, Turkey; gizemkahramann@gmail.com; 2 IMDEA Materials Institute, C/Eric Kandel, 2, Getafe, 28906 Madrid, Spain; deyi.wang@imdea.org; 3 Macromolecular Chemistry Department, Technische Universität Darmstadt, 64287 Darmstadt, Germany; J.vonIrmer@MC.tu-darmstadt.de; 4 Organic Macromolecular Chemistry, Saarland University, Campus Saarbrücken C4 2, 66123 Saarbrücken, Germany; 5 Institute of Inorganic Chemistry, Leipzig University, 04103 Leipzig, Germany

**Keywords:** carboranes, phosphonate esters, block copolymers, ROMP, hybrid materials

## Abstract

Grubbs-catalyzed ring-opening metathesis polymerization (ROMP) of carborane- and phosphonate-containing monomers has been used for the generation of hybrid block copolymers. Molecular weights with *M*_n_ of 50,000 g/mol were readily obtained with polydispersity index values, *Đ*, between 1.03–1.08. Reaction of the phospha ester and carborane substituted oxanorbornene block copolymer with trimethylsilyl bromide led to a new polymer with phosphonic acid functionalities. In application studies, the phospha-carborane functionalized block polymer was tested as heat resistance material. Thermal stability was investigated by thermal gravimetric analysis (TGA) and microscale combustion calorimetry (MCC) analysis. Thermal treatment and ceramic yield under air were directly correlated to the carborane content of the block copolymer. However, phosphorus content in the polymer was more crucial for the char residues when heated under nitrogen atmosphere. The peak heat release rate (PHRR) increased as the number of phosphonate functionalities increased. However, corresponding phosphonic acid derivatives featured a lower heat release rate and total heat release. Moreover, the phosphonic acid functionalities of the block copolymer offer efficient chelating capabilities for iron nanoparticles, which is of interest for applications in biomedicine in the future. The complexation with iron oxide nanoparticles was studied by transmission electron microscopy (TEM) and inductively coupled plasma mass spectrometry (ICP–MS).

## 1. Introduction

The development of hybrid organic–inorganic building blocks is of growing interest. These materials are a typical example of natural systems such as crustacean shells, bone, and tooth tissues. Incorporation of well-defined inorganic clusters inside a comparably soft polymer matrix allows for the development of innovative compounds possessing unusual features for industrial applications [1]. Nanostructures derived by the self-assembly of functional block copolymer is one feasible approach for the preparation of remote-switchable materials at the nanometer length scale [2,3,4,5]. In the last decades amphiphilic block copolymers have attracted a lot of attention because of their unique possibility for self-organization forming micelles or vesicles in selective solvents based on the solubility of their hydrophobic and hydrophilic block segments [6,7,8,9]. Many applications arose from these materials, e.g., in lithography, drug delivery, magnetic nanoparticle stabilization, and, especially, separation technologies with a variety of potential applications [4,6,7,8,9,10,11,12,13,14,15]. Living and controlled polymerizations techniques provide access to polymers and block copolymers featuring narrow molecular weight distributions with controllable molecular weights and defined constitutions. Ring opening metathesis polymerization (ROMP) is one of the living polymerization techniques and can be applied to the preparation of well-defined block copolymers [16,17]. Exploiting the precise control over the polymer chains, as offered by ROMP, the obtained block copolymers can undergo self-assembly via switching capabilities between hydrophobic and hydrophilic states [11,18,19,20,21,22]. For example, multiresponsive pH-dependent self-assembly and further metal ion complexation was explored via the exchange of Cu(II) with Fe(II) ions in the polymer networks [23].

Carboranes are a type of polyhedral inorganic cluster family consisting of ten B-H BH and two C-H fragments and they exist as three isomers (*o*-, *m*-, and *p*-carborane) [24]. Carborane anions produced by organolithium or Grignard can be used for further formation of a variety of derivatives in synthetic carborane chemistry [24,25]. Carborane possesses a highly polarizable σ-aromatic character, which has been exploited in the development of several material systems having aggregation-induced emission (AIE) properties [26] and neutron capture characteristic via generation of α-particles and ^7^Li nuclei through the nuclear reaction of the ^10^B atom with low-energy thermal neutrons [27]. Carboranes have also been known for their use as heat-resistant materials [28,29,30]. The protective char layer via the oxidation of boron to B_2_O_3_ prevents burning of the material via successful trapping of combustible gases and exclusion of oxygen [31]. Application in different fields such as boron neutron capture therapy (BNCT) [24,32,33], precursors for ceramics [34], and optoelectronics [35] have been of growing interest in recent years. There are few studies on the carborane cluster containing block copolymers synthesized via ROMP. Malenfant et al. synthesized an organic–inorganic hybrid block copolymer, polynorbornene–*block*–polynorbornenedecaborane (PNB–*b*–PDB) to prepare nanostructured ceramic materials [36]. After evaporation of the solvent, self-assembly of the hybrid was observed leading to well-defined morphologies on the nanometer length scale. The Coughlin group synthesized amphiphilic carborane-containing block copolymers and investigated the self-assembly into micelles in water [20]. It was observed that the resulting block copolymer forms micelles with an average hydrodynamic radius (R_h_) of 41 nm.

Phosphorus containing polymers have been of growing interest in a variety of applications such as dental adhesives, [37] flame retardants [38,39], and ligands in various organic–inorganic frameworks and hybrid materials [40]. Synthesis of phosphorus-containing monomers and block copolymers thereof are of growing interest in fields of biomedicine, ref. [41] as they are biodegradable as well as blood-compatible showing strong interactions with dentin or bones [42]. In a recent review, the synthesis and radical (co)polymerization of phosphorus-containing monomers via “living”/controlled radical polymerization was highlighted [43]. Specifically, such block copolymers consisting of phosphorus-bearing moieties promoted considerable cell adhesion [44] while featuring a low cytotoxicity [45]. The affinity of phosphorus, phosphonates, and phosphonic acid derived anions for metals and metal oxides enhances the use of these materials as surface modifiers [46]. Compounds bearing phosphonic acids have been used to efficiently coat the surface of iron nanoparticles [47]. Boyer and Davis synthesized well-defined diblock copolymers of poly(oligoethylene glycol) acrylates featuring different anchoring groups, comprised of phosphonic acid, carboxylic acid, and glycerol groups in order to investigate the effect of these functional groups on the size, colloidal stability, crystallinity, and transverse relaxivity properties of the iron oxide nanoparticles [48]. It was observed that the copolymers containing phosphonic acid groups lead to the highest grafting density (0.192 ± 0.01 nm^−2^) and complex more strongly to iron compared to the copolymers with carboxylic acid and glycerol moieties. Marty et al. used poly(ethylene glycol)–poly(vinylphosphonic acid) block copolymers for the stabilization of iron oxide nanoparticles [49]. Additionally, the complexing abilities of poly(vinylphosphonic acid) and polyacrylic acid blocks were compared and improved complexation was observed for the phosphonic acid groups compared with the carboxylic acid and high colloidal stability was observed over several weeks. There are a few studies on the phosphorus-containing type block and/or random copolymers obtained by ROMP [18,50,51,52,53]. Tew and Eren suggested an approach for the synthesis of phosphonic acid-type amphiphilic block copolymers derived from ROMP using Grubbs’ third generation catalyst [18]. It was observed that the micelle dimensions had a broad range and were dependent on the composition of the block copolymers. Jannasch et al. synthesized phosphonate homopolymers, and diblock and triblock copolymers based on poly(norbornene imide)s with low polydispersity (*Đ* = 1.09 − 1.32) [51]. Polymers were also stable up to 240 °C under nitrogen and 160 °C in air. Zhang and Li reported significant self-extinguishing ability of phosphorus containing polymers, and more char residue was obtained at 600 °C preventing combustion [50]. The authors also showed that the phosphonate-functionalized polymer is a promising negative photoresist.

**Scheme 1:**
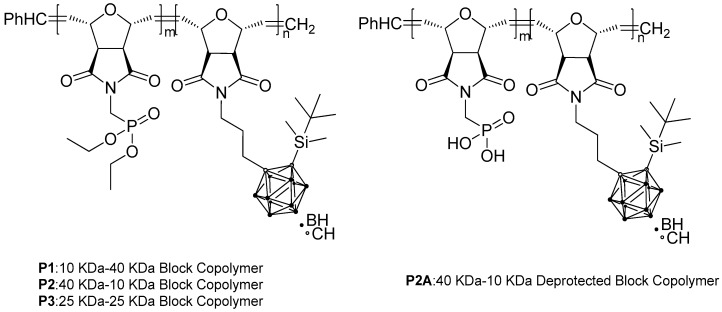
Block copolymers prepared and used in this study.

To the best of our knowledge, the combination of carborane- and phosphorus-functionalized polyoxanorbornene block copolymers has not been developed and investigated until now. The functionalization of organic polymers with carborane and phosphorus offers opportunities to obtain materials with chemical resistance, metal chelation capabilities, and enhanced thermomechanical properties. Moreover, the block copolymer architecture is of interest because of its (block-selective) binding capabilities to various surfaces, for instance metals and metal oxides, as well as for structure formation like micellation, vesicle formation, or microphase separation on the nanometer length scale. Here, we describe such hybrid materials based on carborane- and phosphorus-containing block copolymers obtained via ROMP (Scheme 1) for the first time. These polymers provide multiple functionalities such as (i) amphiphilic carboranes, which can aggregate in aqueous solution to form nano- or microstructures, (ii) carborane-phosphorus-bearing polymer as a flame retarder, and (iii) functional moieties for metal oxide surface coatings, for instance for magnetic nanoparticles. Thermal properties of the block copolymers are investigated by TGA (thermal gravimetric analysis) under N_2_ or in air and by microscale combustion calorimetry (MCC) analysis. The chelating efficiency of polymers bearing phosphonate and phosphonic acid groups towards iron nanoparticles were investigated by means of transmission electron microscopy (TEM) and inductively coupled plasma mass spectrometry (ICP–MS).

## 2. Experimental Section

### 2.1. Materials

Furan, maleimide, diethyl (hydroxy methyl) phosphonate, triphenylphosphine, diisopropyl azodicarboxylate (DIAD), trimethylene oxide, *n*-butyllithium solution (2.5 M in *n*-hexane), *tert*-butyldimethylsilyl chloride, bromo trimethylsilane, ethyl vinyl ether, tetrahydrofuran (THF), petroleum ether, diethyl ether, dichloromethane, and methanol were purchased from Sigma Aldrich and used as received. Ethyl acetate, THF, and *n*-hexane were distilled by conventional techniques. Grubbs’ second generation catalyst was purchased from Sigma Aldrich (Sent Louis, MO, USA). All other reagents including buffers and salts were obtained from Sigma Aldrich (Sent Louis, MO, USA). *o*-Carborane was obtained from Katchem, Prague, Czech Republic. All synthesis steps were conducted under a nitrogen atmosphere using a Schlenk line.

### 2.2. Methods

^1^H NMR, ^13^C NMR, ^11^B NMR, and ^31^P NMR spectra were recorded using a Bruker Avance III 400 MHz spectrometer. Gel permeation chromatography (GPC) analyses were performed with column at 50 °C. 0.01 M LiBr/DMF was used for the mobile phase and toluene was used for the flow marker. Flow rate was 0.7 mL/min. A PerkinElmer Spectrum One spectrometer was used for attenuated total reflectance Fourier transform infrared (ATR–FTIR) spectroscopy. Microscale combustion calorimetry (MCC, Fire Testing Technology, UK) was used for the MCC measurement according to ASTM D7309. Size analysis of polymer-coated nanoparticles in their dry form was conducted by evaporating a drop of an aqueous solution of nanoparticles on a carbon film coated 300 mesh Cu grid (Pacific Grid Tech, USA). Images were acquired using transition electron microscopy (TEM) with an accelerating voltage of 5 kV on a LVEM5 electron microscope (Delong Instruments, USA). TEM samples were prepared by dropping of the coated nanoparticles on a carbon-coated copper grid and then left to dry overnight. SII Nanotechnology, TG/DTA 6300 Series was used for thermal analysis. The thermal stability of polymers was investigated between room temperature and 600 °C under nitrogen and air with a heating rate of 25 °C/min.

### 2.3. ROMP (Ring Opening Metathesis Polymerization) Block Copolymers

The block copolymers were synthesized by the ROMP pathway by the addition of Grubbs’ 3rd generation catalyst. In order to successfully obtain the blocks, phosphonate-substituted *exo*-oxanorbornene imide (0.5 g, 1.59 mmol) was stirred at room temperature with Grubbs’ 3rd generation catalyst (0.011 g, 0.5 mol%) in dry dichloromethane for 1 h under nitrogen atmosphere. Once the first block polymer was determined by the GPC technique, silyl-protected oxanorbornene imide carborane-containing oxanorbornene was added into the reaction mixture after first being dissolved in dry dichloromethane. The reaction was completed after the addition of 30% ethyl vinyl ether and stirring in the reaction flask for 30 min. The products were dried in a vacuum oven and then kept under nitrogen.

### 2.4. Deprotection of ROMP (Ring Opening Metathesis Polymerization) Block Copolymer P2

The deprotection of block copolymer **P2** was performed by dissolving 0.1 g of polymer in 10 mL dry dichloromethane followed by the addition of 2 mL bromotrimethylsilane at room temperature. The reaction was stirred under nitrogen at 40 °C for 4 h, then 10 mL methanol was added to the reaction mixture after cooling down to room temperature. It was stirred for another hour and the final product, phosphonic acid derivative, **P2A**, was obtained after washing with diethylether.

### 2.5. Preparation of Polymer Coated-Nanoparticles

In the first part of the study, bare iron nanoparticles were synthesized using the co-precipitation method. Eighty milliliters of distilled water was placed into a jacketed glass reactor and deaerated using nitrogen gas for 30 min while stirring with a mechanical stirrer (200 rpm) at 60 °C. After deaeration, 0.28 g of FeCl_3_ and 0.24 g of FeSO_4_.·7H_2_O was added to the reactor and left stirring for 15 min. Then, NaOH solution (0.51 g dissolved in distilled H_2_O) was added to the reactor and nitrogen gas was stopped. The solution turned black immediately after base addition and stirring continued for 30 min. Then the reaction mixture was transferred into a falcon tube and centrifuged at 5000 rpm for 5 min. The obtained bare iron oxide particles were washed with acetone three times and dried using a lyophilizer.

For the preparation of polymer coated nanoparticles, a certain amount of deprotected block copolymer was sonicated in 1 mL DMSO for 6 h at room temperature after adding 5% iron oxide nanoparticles to a scintillation vial. Then a dialysis membrane was used to get rid of uncoated nanoparticles on the surface of the polymer. The ICP results confirmed the amount of nanoparticles on the polymer surface and TEM images were taken.

## 3. Results and Discussion

Cyclic oxanorbornene monomers suitable for metathesis bearing phosphonate (monomer 1) or carborane units (monomer 2) were synthesized and used for the synthesis of diblock copolymers via ROMP. Three series of block copolymers, **P1**, **P2**, and **P3**, were synthesized, keeping the molecular weight constant (*M*_n,th_ = ~50,000 g/mol) with varying monomer compositions by weight ratios (1:4, 4:1, and 1:1) to investigate the structure-thermal property relationship for the carborane and phosphorus contents. Cleavage of the phosphonate groups with trimethylsilylbromide (TMSBr) resulted in phosphonic acid-bearing amphiphilic block copolymer, **P2A**.

### 3.1. Monomer Synthesis

Phosphonate-substituted *exo*-oxanorbornene imide (monomer 1) was synthesized and characterized according to the literature (see Scheme S1, electronic supplementary information (ESI)) [18]. Characteristic signals for the phosphonate units were observed at 1.32 and 4.16 ppm in the ^1^H NMR spectra (Figure S1, ESI). The ^31^P NMR spectrum (Figure S2, ESI) shows only one singlet at 18.4 ppm. 16.3 and 62.9 ppm in the ^13^C{^1^H}- NMR spectra (Figure S3, ESI), which can be assigned to the ethyl group (O=P-OCH_2_CH_3_). A literature procedure was also used for the synthesis of silyl-protected carborane-containing oxanorbornene, monomer 2 (see Scheme S2, ESI) [20]. In the first part of the study, silyl protection and hydroxyl functional carborane precursor was synthesized and characterized by ^1^H, ^13^C, and ^11^B NMR techniques (Figures S4–S9, ESI). Furthermore, silyl protected carborane-containing oxanorbornene monomer (monomer 2) was confirmed by ^1^H and ^13^C{^1^H}- NMR spectra (Figures S10 and S11, ESI). Carborane was also observed by ^11^B-NMR and indicated 4 peaks as expected (Figure S12, ESI). Characteristic boron peaks were observed at 0.35 ppm (1B), −3.85 ppm (1B), −7.47 (2B), and −10.38 ppm (6B). 

### 3.2. Synthesis of Carborane- and Phosphorus-Containing Block Copolymers

The synthesis of the block copolymers is given in Scheme S3 (see ESI). The first block of the polymer was obtained by reaction of the first monomer 1 using Grubbs’ 3rd generation catalyst as an initiator in dry dichloromethane. Monomer conversion was monitored by TLC and ^1^H NMR spectroscopy, and was typically completed after 30 min. After full conversion of the first monomer, the second monomer 2 was added to the reaction mixture. The polymerization was quenched by adding ethyl vinyl ether and then the reaction mixture was poured into an excess of petroleum ether. TLC analysis indicated complete conversion. The polymers were dried in vacuum for 24 h. Characterization with respect to constitution, molar mass, and thermal degradation of the block copolymers were done using NMR spectroscopy, gel permeation chromatography (GPC), and thermal gravimetric analysis (TGA). The completion of polymerization was shown by disappearance of the characteristic olefin protons of the monomers at 6.5 ppm and the appearance of the backbone double bond (cis/trans) signals between 5.5 and 6.0 ppm in the ^1^H NMR spectra.(see the ^1^H NMR spectra of **P1**, **P2**, and **P3** in Figures S13, S18, and S22, respectively, and ^13^C{^1^H}, ^31^P NMR and ^11^B spectra of **P1**, **P2** and **P3** were also reported in Figures S15–S25, ESI).

Stereoisomers were analyzed by comparing the ratio of signals c_cis_ and c_trans_. It was observed that the signal of cis protons showed a higher intensity than the trans proton peaks (ratio ~60%/40%, Table 1). The distribution of molecular weight and the polydispersity index (*Đ*) of the block copolymers were determined by size exclusion chromatography (SEC) in DMF using poly(methyl methacrylate) (PMMA) as a standard. In this study, according to SEC results, narrow molecular weight distributions with a *Đ* between 1.03 and 1.09 were observed. *M*_n_ of the respective polymers was also calculated by end group analysis of the purified polymers. Styrenic end groups coming from the carbene moiety of the catalyst were visible at ca. δ ~7.20–7.50 ppm and were used to calculate the degree of polymerization. Table 1 shows that the calculated molecular weight of the polymers is close to the targeted molecular weight. The ratio of the number of phosphonate repeat units (m, Table 1) to the number of carborane repeat units (*n*, Table 1) was analyzed by ^1^H NMR spectroscopy (i.e., four protons for the phosphonate group (-P-OCH_2_-) at 4.13 ppm and six protons at 0.33 ppm for the SiMe_2_ group. The feed ratios of other copolymers, and targeted and observed molecular weight specified by SEC as well as calculated by using NMR [18] are shown in Table 1. In a typical example, the molecular weights of the first (phosphonate part) and second repeating units (carborane part) in **P3** were 315 and 463 g/mol, respectively; as the target repeating units ratio was 79.4:53.9 (polymer **P3**, Table 1), then the theoretical number average molecular weight was (*M*_n,th_) = (315 × 79.4) + (436 × 53.9) = 50,000 g/mol. The observed molecular weight of **P3** using styrenic end group analysis [18] via NMR was 54,081 g/mol. However, caution should be taken when the NMR quantification is applied. The integration values of the ^1^H NMR peaks were found to be strongly dependent on the solvent used. The actual peak intensities and relative integrations are being represented correctly only if no micelle formation occurs. To investigate the solvent effect, the ^1^H NMR spectra of the most hydrophobic polymer (**P1**) were analyzed using the solvents DMSO-d_6_ and CDCl_3_. The ^1^H NMR spectra obtained in DMSO-d_6_ indicated that the integration value of the methyl protons (-Si-CH_3_) in Figure S14 (ESI) was too low due to the silyl units being included in the core of the micelle. This disappearance is in agreement with the formation of core-shell micelles in which the hydrophobic block is hidden in the micelle core in a non-solvated state [18]. Nevertheless, using CDCl_3_, the methyl protons (-Si-CH_3_) can easily be observed in the ^1^H NMR spectrum (Figure S13, ESI).

### 3.3. Conversion of the Phosphonate Ester to Phosphonic Acid, P2A

Phosphonic acids exhibit many interesting properties such as acting as chelates for metal ions or proton conductivity in fuel cell membranes under anhydrous conditions. They also enhance the fire retardancy performance of material and are furthermore useful in biomedical area, e.g., in dental composites with a good chelation efficiency to dentin.

**Figure 1:**
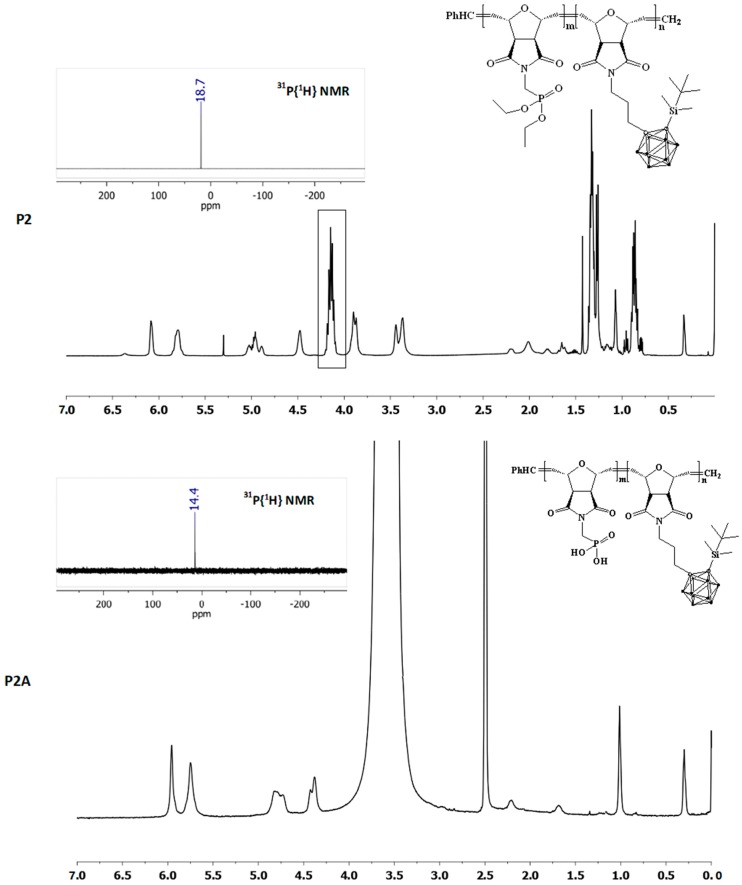
^1^H NMR spectra before and after conversion of the phosphonate ester groups, polymers **P2** and **P2A** (spectrum **P2** obtained in CDCl_3_, spectrum **P2A** in DMSO-d_6_). The insets are corresponding ^31^P{^1^H}NMR spectra of the respective polymers.

Here, we chose the block copolymer with the highest number of phosphonate ester units, **P2**, for conversion to the corresponding phosphoric acid using TMSBr followed by hydrolysis with methanol/dichloromethane (3:1, v:v). After purification **P2A** was obtained as a rubbery soft solid. P2A was characterized by NMR spectroscopy (Figures S26 and S27, ESI). Particularly, ^31^P{^1^H}NMR was the most informative technique. Disappearance of the phosphonate ester peak at 4.13 ppm in the ^1^H NMR spectrum was observed after cleavage, and the ^31^P{^1^H} NMR spectrum showed a shift from 18.7 to 14.4 ppm (Figure 1, inset spectra), corresponding to the phosphonate ester and phosphonic acid, respectively.

**Figure 2:**
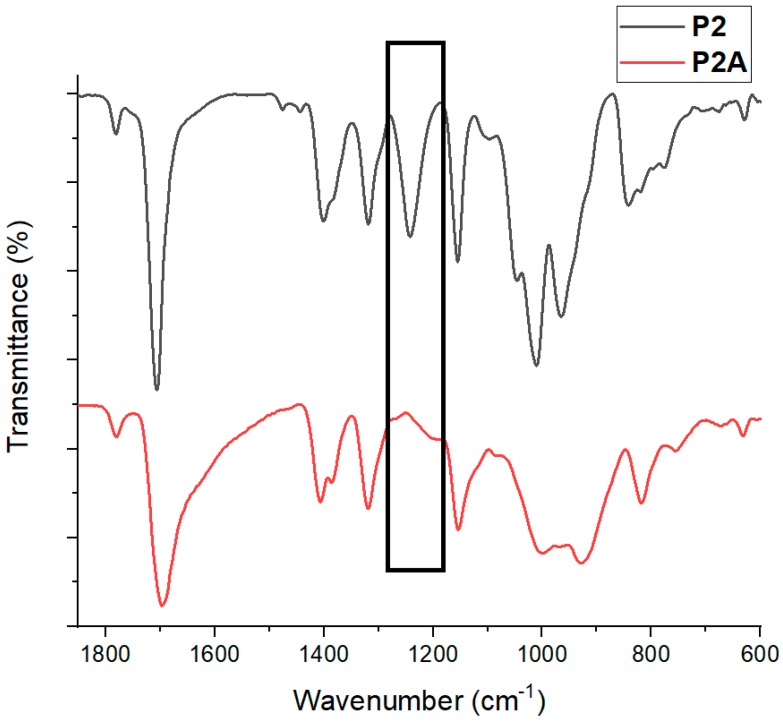
FT-IR spectra of **P2** and **P2A**.

Figure 2 shows the FT–IR spectrum of block copolymer **P2** with the vibrations at 1260 cm^−1^ and 1110 cm^−1^ indicating the phosphonate groups (P=O) and (P-O) which disappeared after conversion to the corresponding phosphonate acid (**P2A**).

### 3.4. Thermal Properties of the Polymers

**Figure 3:**
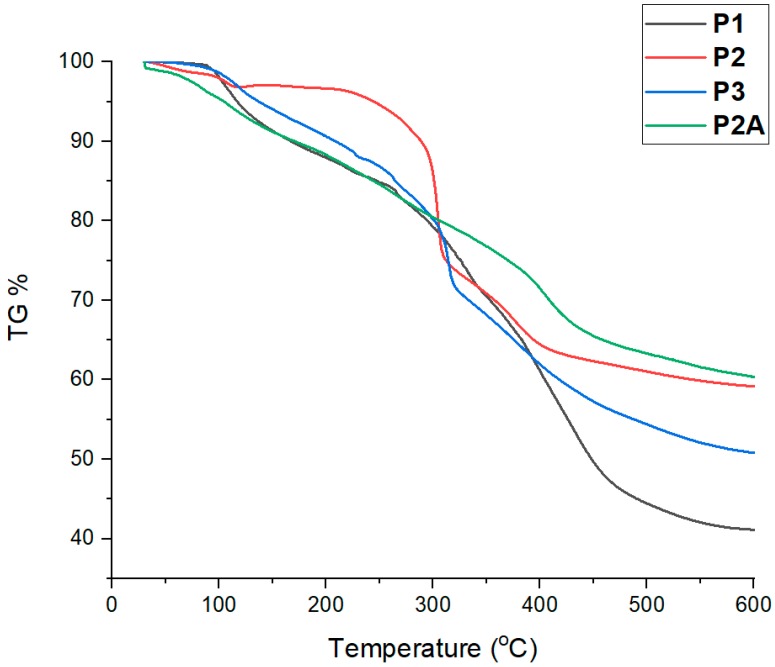
Thermal gravimetric analysis of phosphonate ester- or phosphoric acid- and carborane-bearing block copolymers under nitrogen atmosphere.

**Figure 4:**
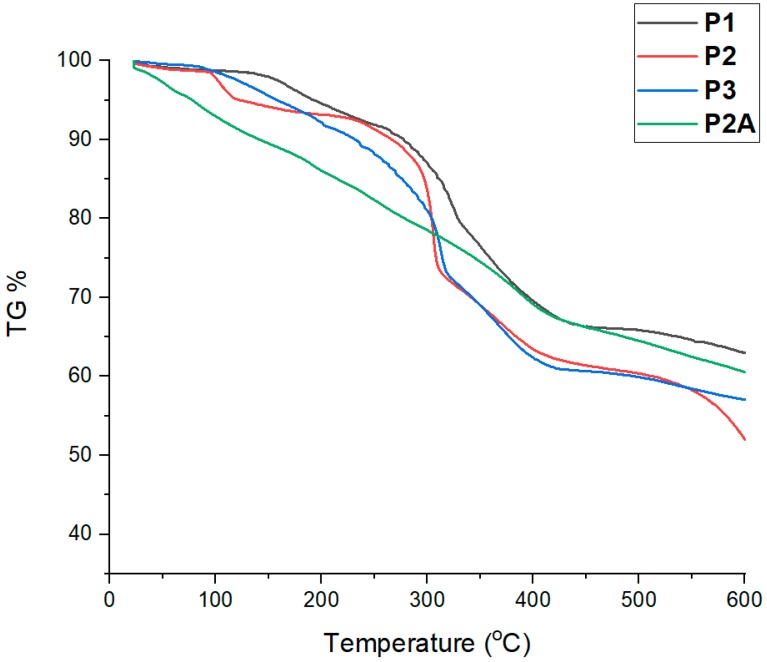
Thermal gravimetric analysis of phosphonate ester- or phosphoric acid- and carborane-bearing block copolymers in air.

The thermal properties of the block copolymers **P1**, **P2**, **P3**, and **P2A** were investigated by TGA. TGA was conducted under N_2_ as well as in air (heating rate 10 °C min^−1^, *T* range 25–600 °C) (Figure 3 and Figure 4). It was expected that a higher number of phosphonate ester groups might enhance the char yield. Before the analysis, the phosphorus and carborane contents of each polymer was calculated by quantitative analysis of each repeating unit. The phosphorus contents of block copolymers **P1**, **P2**, and **P3** can be calculated with the following equation:

(1) $$P\;content\;by\;weight=\frac{\left[\left(\frac{MW\;of\;phosphonate\;block}{MW\;of\;monomer\;1}*31\right)*100\right]}{MW\;of\;polymer\;chain}$$

For example, **P1** with a molecular weight of 56,756 g/mol has 31.75 repeating units containing phosphonate ester groups in its backbone. The molecular weight of each repeating unit is 315 g/mol and each unit contains one phosphorus atom with a molecular weight of 31 g/mol, in total 2.66 weight% P. Accordingly, the calculated weight% P in **P2** and **P3** is 7.04% and 5.86%, respectively. Polymer **P2** which had the highest phosphorus content resulted in 59.3% char residue under nitrogen (Table 2).

In the first part of the study, the thermal stabilities of the phosphonate and carborane based ROMP homopolymers with a theoretical molecular weight of 50,000 g/mol were compared. It was observed that phosphonate homopolymer was more stable revealing a char residue of 52.2% under nitrogen atmosphere. However, carborane based homopolymer resulted in 32.2% char residue under the same conditions. Phosphonic acid derivatives of the corresponding phosphonate ester also yielded higher char residue. It is worthy to mention that pyrolysis under inert atmosphere is of interest (i) for the preparation of ceramic hybrid materials and (ii) for gaining insights into the structure-pyrolysis relationship and flame retardancy. Compared to these findings, char residue of carborane homopolymer increased to 40% under air atmosphere. The thermal resistance of the phosphonic acid-based homopolymer was higher than the corresponding phosphonate ester homopolymer under air.

Polymer **P1** with the highest carborane content gave a char residue of 41.2%. However, increasing the carborane content in the polymer backbone resulted in an increased char residue in air due to capture of oxygen by the boron cluster to form a B_2_O_3_ layer. In air, the char yield of **P2** dropped to 48.4%, while for **P1** it increased to 62.4% at 600 °C. An excellent char yield was observed for the polymer **P2A** with phosphonic acid both in air (60.6%) and nitrogen atmosphere (60.4%) at 600 °C. The initial weight loss at around 100 °C for the **P2A** is attributed to the release of physically bound water [54]. Phosphonic acid residues are capable of capturing moisture, which appeared under storage conditions and weight loss in the temperature range of 100–200 °C can stem from the residual moisture and the dehydration of water in the polar polymer matrix. However, the loss of water molecules continues at higher temperatures through the formation of anhydrides (P–O–P bonds) during polymer degradation. Nevertheless, further detailed combustion analyses must be conducted to understand the mechanism of fire retardancy.

MCC is used for the analysis of the flammability of materials. The analysis give the values of the average heat release rate (HRR), peak heat release rate (PHRR), total heat release (THR), and temperature at peak heat release rate (TPHRR). HRR shows the fire risk of the materials; the higher the HRR value, the higher the combustibility of the material. Figure S28 (ESI) shows the MCC curves for HRR against temperature. It was observed that an increased carborane content in **P1**, **P2**, and **P3** led to a reduced PHRR. The PHRR increases as the number of phosphonate groups increases in the following order **P2** > **P3** > **P1**; i.e., PHRR values were 275.5 (**P2**), 225.6 (**P3**), and 74.5 W/g (**P1**). The introduction of phosphonic acid moieties in **P2A** resulted in a much lower PHRR value (13 W/g) when compared to the phosphonate ester block copolymers **P1**, **P2**, and **P3**. This suggests that the flammability of phosphonic acid-bearing polymers is considerably lower than that of the corresponding phosphonate ester-bearing polymer under the same combustion conditions. It is also well known that most P–N compounds leave considerable amounts of char residues after combustion. In summary, the synergistic effect of phosphorus and nitrogen besides the presence of silicon and boron clusters leads to enhanced thermal properties of the polymers used in this study.

### 3.5. Stabilization of Magnetic Fe_3_O_4_ Nanoparticles with Block Copolymer P2A

**Scheme 2:**
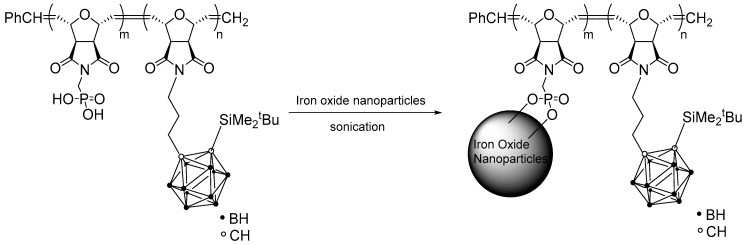
Synthetic scheme for the iron oxide nanoparticles coating by using polymer **P2A**.

**Figure 5:**
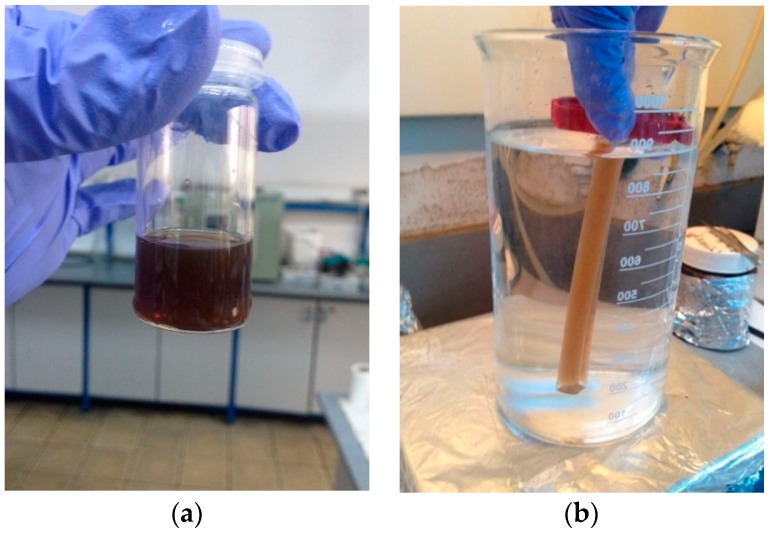
Iron oxide nanoparticles coated with phosphonate-bearing block copolymer **P2A**. (**a**) P2A-iron oxide nanoparticles in DMSO, before dialysis; (**b**) P2A-iron oxide nanoparticles in water, after dialysis.

Magnetic iron oxide nanoparticles represent a class of inorganic nanomaterials that contribute significantly to the field of microelectronics, magnetic data storage, biosensors, etc. Their unique physical properties, including high surface area to volume ratios and superparamagnetism, are also useful attributes in medical applications such as magnetic resonance imaging (MRI), drug and gene delivery, or tissue engineering. It is important to stabilize the nanoparticles in a suitable way to prevent further agglomeration and phase separation. Herein, we report the design and preparation of iron oxide nanoparticles by using the block copolymer **P2A**, which contains phosphonic acid groups that are known to bind strongly to the iron oxide surface as ligands [55,56]. After dissolving polymer **P2A** in DMSO, bare iron oxide nanoparticles were added to the solution and sonicated for 7 h. Scheme 2 shows the synthetic procedure for the coating process. Some precipitated aggregates were removed by centrifugation at 2500 rpm for 5 min and dialysis against water for one week. When the polymer coordinates at the surface of the iron oxide nanoparticles, the color of the DMSO solution turns from red to pale yellow after dialysis against water (see in Figure 5). The product, surface-coated iron oxide nanoparticles, was collected from the dialysis membrane and characterized by inductively coupled plasma (ICP) mass spectrometry (ICP–MS) and TEM.

The use of phosphonates as ligands for the construction of metal-based polymeric networks has been mentioned in the literature [57]. In a simple quantitative demonstration of the superior binding efficiency of phosphonate to iron oxide nanoparticles compared to phosphonate esters, the iron oxide nanoparticles were suspended in DMSO solution and mixed with the block copolymers **P2**. Obviously, **P2A** is the better ligand, as indicated in Table 3. It was observed that phosphonate ester-bearing polymer **P2** contained 61 ppm iron, and its phosphonic acid analog **P2A** 338 ppm iron (as determined with ICP–MS).

After dialysis against water, the morphology of the nanoparticles coated with polymer was studied by TEM. The coated nanoparticles were simply drop-casted on a carbon-coated copper grid and then left to dry overnight. Aggregation of nanoparticles was observed (Figure S29, ESI). The phosphonic acid groups on the outer shell and carborane moieties forming the inside shell provide good colloidal stability in DMSO, however precipitation occurs in water. It was also observed that polymer-coated nanoparticles agglomerate together in aqueous environments, forming spherical micellar morphologies. The distribution of the nanoparticles was not homogeneous, and the sample showed particle coalescence in the TEM micrographs. Magnetic interaction between nanoparticles and self-assembly via the phosphonic acid groups can result in vesicles or micelles and cause the observed large aggregation in aqueous environments. It is obvious that surface charges can be tailored by the presence of different surface-active compounds for iron oxide (such as phosphonates). However, the underlying mechanism for the preorganization mechanism induced by the addition of iron nanoparticles to a block copolymer solution bearing phosphonate ester or phosphonic acid groups (**P2A**) leading to the formation of surface coating should be further investigated. In situ coprecipitation for a high load of nanoparticles using Fe^2+^ or Fe^3+^ salts from aqueous solutions in the presence of phosphonic acid-bearing block copolymers is presently being studied. Inverted micelle formation in tetrahydrofuran/water mixtures is also another strategy for achieving high loads of iron oxide nanoparticles inside the micelle. This study is also underway.

## 4. Conclusions

In this study we described the synthesis of amphiphilic block copolymers with narrow molecular weight distributions prepared by ROMP of carborane-containing oxanorbornene and phosphorus-containing oxanorbornene monomers. Molecular weights (*M*_n_) of 50,000 g/mol were readily obtained with polydispersity index values between 1.03 and 1.08. Conversion of phosphonate ester groups to phosphonic acid resulted in an amphiphilic polymer **P2A**. Thermal analysis showed that the char yield obtained by TGA in air was directly related with the carborane level in the block copolymer. For example, **P1** had the highest carborane content and had a 62.4% char yield at 600 °C. However, the phosphonate units played a major role in TGA under N_2_ with a char yield of 59.3% related with the highest phosphorus content. TGA of the phosphonic acid derivative resulted in a high char yield of 62.3% in both air and nitrogen. MCC studies showed that the PHRR values decreased as the ratio of the carborane moieties increased in the block copolymers. However, introducing the phosphonic acid group resulted in the lowest heat release rate and incombustibility properties of the polymer. These amphiphilic block copolymers could have potential applications as nanoscopic templates where high heat resistance is necessary.

The amphiphilic polymer **P2A** was used to chelate iron oxide nanoparticles. One potential application of these novel materials could be magnetically induced delivery of carborane-containing polymers in boron neutron capture therapy (BNCT) for the treatment of cancer. Mimicry of nature with well-defined clusters of inorganic and organic hybrid systems is a topic of growing interest. Both phosphorus and carborane derivatives are typical examples that can be further used for such modifications.

## Figures and Tables

**Table 1:** Summary of the characterization of the block copolymers **P1**, **P2**, and **P3**.

Polymer	m ^a^	n ^b^	Mol Ratio Th (m/n) ^c^	Mol Ratio Obs (m/n) ^d^	*M*_n/block_ Th (g/mol) ^e^	*M*_n/block_ NMR (g/mol) ^f^	*Đ* ^g^	Cis/Trans Ratio ^h^
**P1**	31.75	86.33	0.37	0.42	50,000	56,756	1.06	1.48
**P2**	127	21.59	5.88	6.48	50,000	55,102	1.09	1.43
**P3**	79.365	53.95	1.47	1.59	50,000	54,081	1.03	1.44

^a^: Theoretical degree of polymerization (DP) of monomer 1. ^b^: Theoretical DP of monomer 2. ^c^: Theoretical molar ratio of monomers. d: Calculated molar ratio using NMR. ^e^: Theoretical number-average molecular weight. ^f^: *M*_n_ calculated by NMR. ^g^: Polydispersity index (Đ) determined by SEC. ^h^: cis/trans ratio in the polymer backbone observed by ^1^H NMR spectroscopy.

**Table 2:** Char residues (%) of the block copolymers **P1**, **P2**, **P3**, and **P2A** (determined by thermal gravimetric analysis (TGA) under N_2_ and in air) at 600 °C.

Polymer	Char Residue (under N_2_)%	Char Residue (in Air)%
**P1**	41.2	62.4
**P2**	59.3	48.4
**P3**	51.1	56.8
**P2A**	60.4	60.6

**Table 3:** Iron concentration obtained from the polymer samples ^a.^

Polymer	Fe Amount Before Dialysis	Fe Amount After Dialysis
**P2**	858 ppm	61 ppm
**P2A**	758 ppm	338 ppm

^a^ ICP MS result. Fe LOQ: SHIMADZU ICPE900; 0.1 mg/L, method; 0.5 with 2.6977% relative standard deviation.

## Supplementary Materials

The following are available online at https://www.mdpi.com/2073-4360/11/4/613/s1. Scheme S1. Synthesis of Monomer 1. Scheme S2. Synthesis of Monomer 2. Scheme S3. Synthesis of carbaborane- and phosphonate ester-based polynorbornene polymers. Figure S1. 1H NMR spectrum of phosphonate ester-based monomer (Monomer 1). Figure S2. 31P NMR spectrum of phosphonate ester-based monomer (Monomer 1). Figure S3. 13C{1H} NMR spectrum of phosphonate ester-based monomer (Monomer 1). Figure S4. 1H NMR spectrum of compound A. Figure S5. 13C{1H} NMR spectrum of compound A. Figure S6. 11B{1H} NMR spectrum of compound A. Figure S7. 1H NMR spectrum of compound B. Figure S8. 13C{1H} NMR spectrum of compound B. Figure S9. 11B{1H} NMR spectrum of compound B. Figure S10. 1H NMR spectrum of carbaborane-based monomer (Monomer 2). Figure S11. 13C{1H} NMR spectrum of carbaborane-based monomer (Monomer 2). Figure S12. 11B{1H} NMR spectrum of carbaborane-based monomer (Monomer 2). Figure S13. 1H NMR spectrum of phosphonate ester- and carbaborane-based block copolymer with a theoretical ratio m:n (1:4) in CDCl3. Figure S14. 1H NMR of phosphonate ester- and carbaborane-based block copolymer with a theoretical ratio m:n (1:4) in DMSO-d6. Figure S15. 13C{1H} NMR spectrum of phosphonate ester and carbaborane-based block copolymer with a theoretical ratio m:n (1:4). Figure S16. 31P{1H} NMR spectrum of phosphonate ester- and carbaborane-vbased block copolymer with a theoretical ratio m:n (1:4). Figure S17. 11B{1H} NMR spectrum of phosphonate ester- and carbaborane-based block copolymer with a theoretical ratio m:n (1:4). Figure S18. 1H NMR spectrum of phosphonate ester- and carbaborane-based block copolymer with a theoretical ratio m:n (4:1). Figure S19. 13C{1H} NMR spectrum of phosphonate ester- and carbaborane-based block copolymer with a theoretical ratio m:n (4:1). Figure S20. 31P{1H} NMR spectrum of phosphonate ester- and carbaborane-based block copolymer with a theoretical ratio m:n (4:1). Figure S21. 11B{1H} NMR spectrum of phosphonate ester- and carbaborane-based block copolymer with a theoretical ratio m:n (4:1). Figure S22. 1H NMR spectrum of phosphonate ester- and carbaborane-based block copolymer with a theoretical ratio m:n (1:1). Figure S23. 13C{1H} NMR spectrum of phosphonate ester- and carbaborane-based block copolymer with a theoretical ratio m:n (1:1). Figure S24. 31P{1H} NMR spectrum of phosphonate ester- and carbaborane-based block copolymer with a theoretical ratio m:n (1:1). Figure S25. 11B{1H} NMR spectrum of phosphonate ester- and carbaborane-based block copolymer with a theoretical ratio m:n (1:1). Figure S26. 1H NMR spectrum of phosphonic acid- and carbaborane-based block copolymer with a theoretical ratio m:n (4:1). Figure S27. 31P{1H} NMR spectrum of phosphonic acid- and carbaborane-based block copolymer with a theoretical ratio m:n (4:1). Figure S28. TEM observation of iron oxide nanoparticles coated with phosphonate-based polymer, P2A. Figure S29. HRR curves of polymers from MCC testing.
