# Supplementary material for: Synthesis and Characterization of Phosphorus- and Carborane-Containing Polyoxanorbornene Block Copolymers
Source: Polymers (Basel). 2019 Apr 3;11(4):613. doi: 10.3390/polym11040613 (PMC6523416; doi:10.3390/polym11040613)
Supplement: Supplementary file 1 [file polymers-11-00613-s001.pdf]

Supporting Information

Synthesis and Characterization of Phosphorus- and Carborane-Containing Polyoxanorbornene Block Copolymers

Gizem Kahraman,[1] De-Yi Wang,[2] Jonas von Irmer,[3] Markus Gallei,[3,4]* Evamarie Hey-Hawkins,[5]* and Tarik Eren[1]*

1 Chemistry Department, Yildiz Technical University, Istanbul 34220, Turkey; gizemkahramann@gmail.com
2 IMDEA Materials Institute, C/Eric Kandel, 2, 28906 Getafe, Madrid, Spain; deyi.wang@imdea.org
3 Macromolecular Chemistry Department, Technische Universität Darmstadt, Darmstadt 64287, Germany
4 Organic Macromolecular Chemistry, Saarland University, Campus Saarbrücken C4 2, 66123 Saarbrücken, Germany
5 Institute of Inorganic Chemistry, Leipzig University, Leipzig 04103, Germany; hey@uni-leipzig.de

Received: date; Accepted: date; Published: date

* Correspondence: erentari@gmail.com, hey@uni-leipzig.de m.gallei@mc.tu-darmstadt.de

1. Synthesis

Synthetic pathway for monomer 1 is given in scheme S1, for monomer 2 in scheme S2. Schematic representation of phosphorus-carborane-based block copolymers is given in scheme S3. The phosphonic acid-based derivative is given in scheme S4.

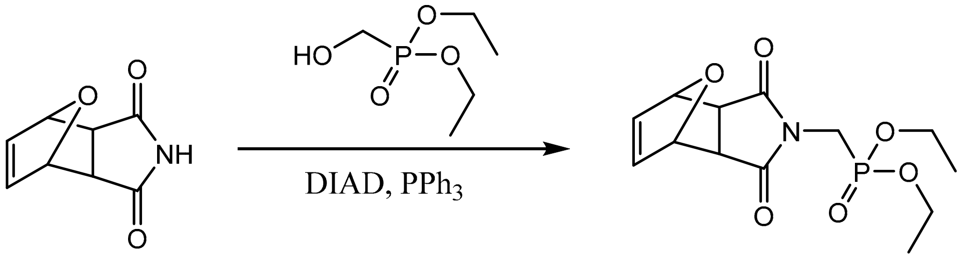

Scheme S1. Synthesis of Monomer 1

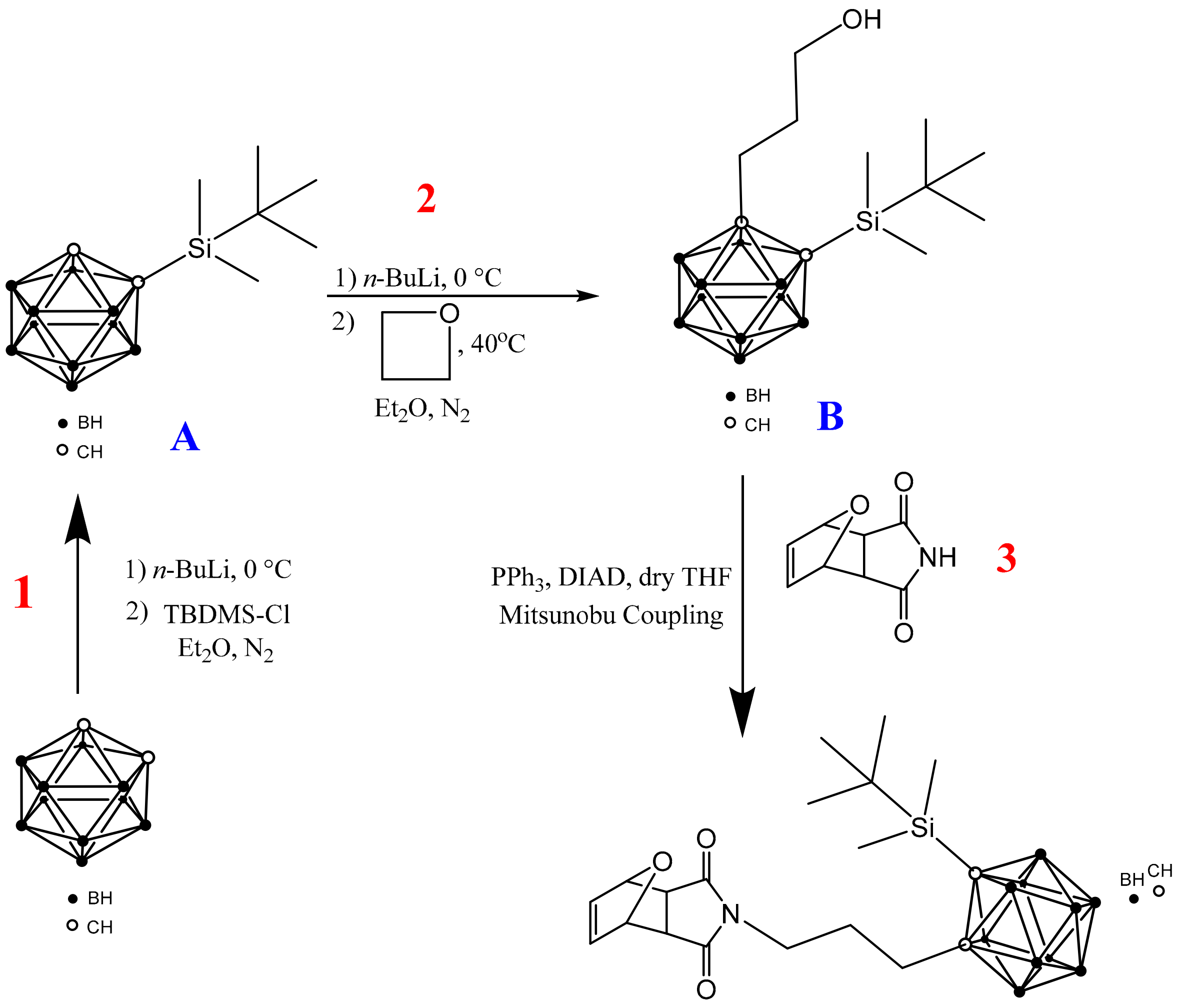

Scheme S2. Synthesis of Monomer 2

Synthesis of Monomer 1

Monomer 1 was prepared according to the literature.[S1]

Synthesis of Monomer 2

Monomer 2 was prepared according to the literature.[S2]

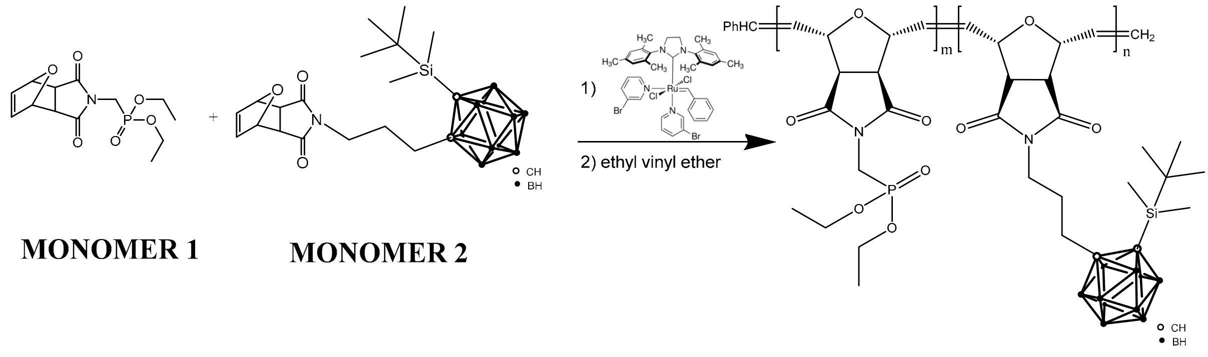

MONOMER 1 **MONOMER 2**

P1:10KDa-40KDa Block Copolymer
P2:40KDa-10KDa Block Copolymer
P3:25KDa-25KDa Block Copolymer

Scheme S3. Synthesis of carbaborane- and phosphonate ester-based polynorbornene polymers

Synthesis of Diblock Copolymers (P1, P2, P3)

Grubbs' third generation catalyst [(H2-Imes)(3-Br-py)2(Cl)2Ru=CHPh] was freshly prepared according to the previously reported procedure.[S3] In a Typical experiment, 0.25 g (0.79 mmol) of monomer 1 was dissolved in CH_2Cl_2 (DCM). The required amount of catalyst was dissolved in CH_2Cl_2 in a separate flask, and then the catalyst solution was added to the monomer solution with vigorous stirring. The final monomer concentration was 0.2 M. After the polymerization of the first block was complete (~1 h, confirmed by ^{1}H NMR spectroscopy or TLC), the required amount of monomer 2 previously dissolved in CH_2Cl_2 was added to the polymerization mixture. The polymerization was allowed to continue for 1 h to completion. The reaction was stopped by the addition of an excess of ethyl vinyl ether while stirring for 30 min.[S1] The polymer was precipitated and washed with petroleum ether, then dried under nitrogen. Block copolymers were dissolved in $CDCl_3$ for NMR characterization.

P1: ^{1}H NMR (400 MHz, $CDCl_3$, ppm) δ: 6.08 (s, 2H), 5.80 (s, 2H), 4.98 (m, 2H), 4.47 (m, 2H), 4.14 (m, 4H), 3.90 (t, 2H), 3.44-3.37 (m, 6H), 2.20 (m, 2H), 1.80 (br t, 2H), 1.28 (t, 6H), 1.07 (s, 9H), 0.33 (s, 6H).

P2: ^{1}H NMR (400 MHz, $CDCl_3$, ppm) δ: 6.08 (s, 2H), 5.79 (s, 2H), 4.96 (m, 2H), 4.48 (m, 2H), 4.15 (m, 4H), 3.90 (t, 2H), 3.44-3.37 (m, 6H), 2.18 (m, 2H), 2.01 (br t, 2H), 1.34 (t, 6H), 1.07 (s, 9H), 0.32 (s, 6H).

P3: ^{1}H NMR (400 MHz, $CDCl_3$, ppm) δ: 6.08 (s, 2H), 5.79 (br s, 2H), 4.98 (m, 2H), 4.47 (br s, 2H), 4.13 (m, 4H), 3.87 (t, 2H), 3.44-3.37 (m, 6H), 2.20 (br s, 2H), 1.90 (br t, 2H), 1.33 (t, 6H), 1.06 (s, 9H), 0.33 (s, 6H).

2. NMR Spectra of All Products

Monomers

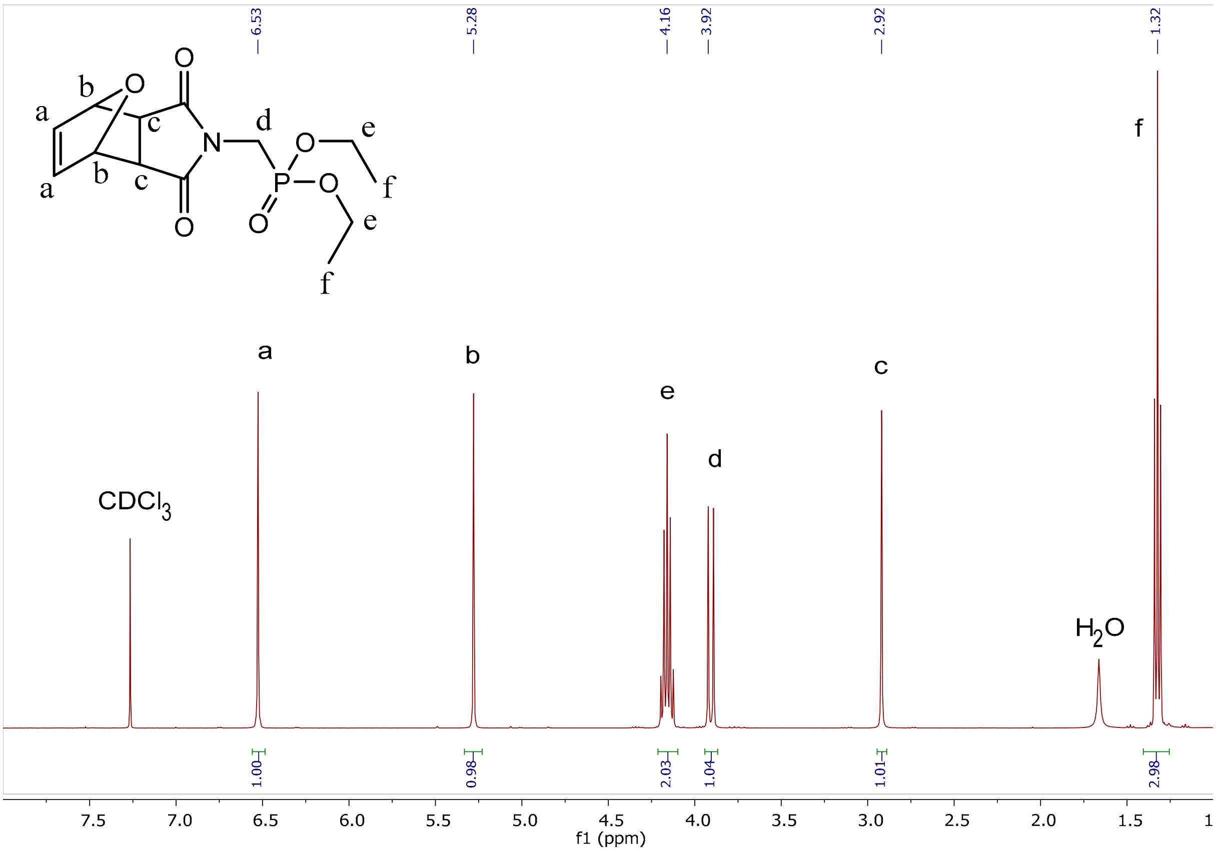

Figure S1. ^{1}H NMR spectrum of phosphonate ester-based monomer (Monomer 1)

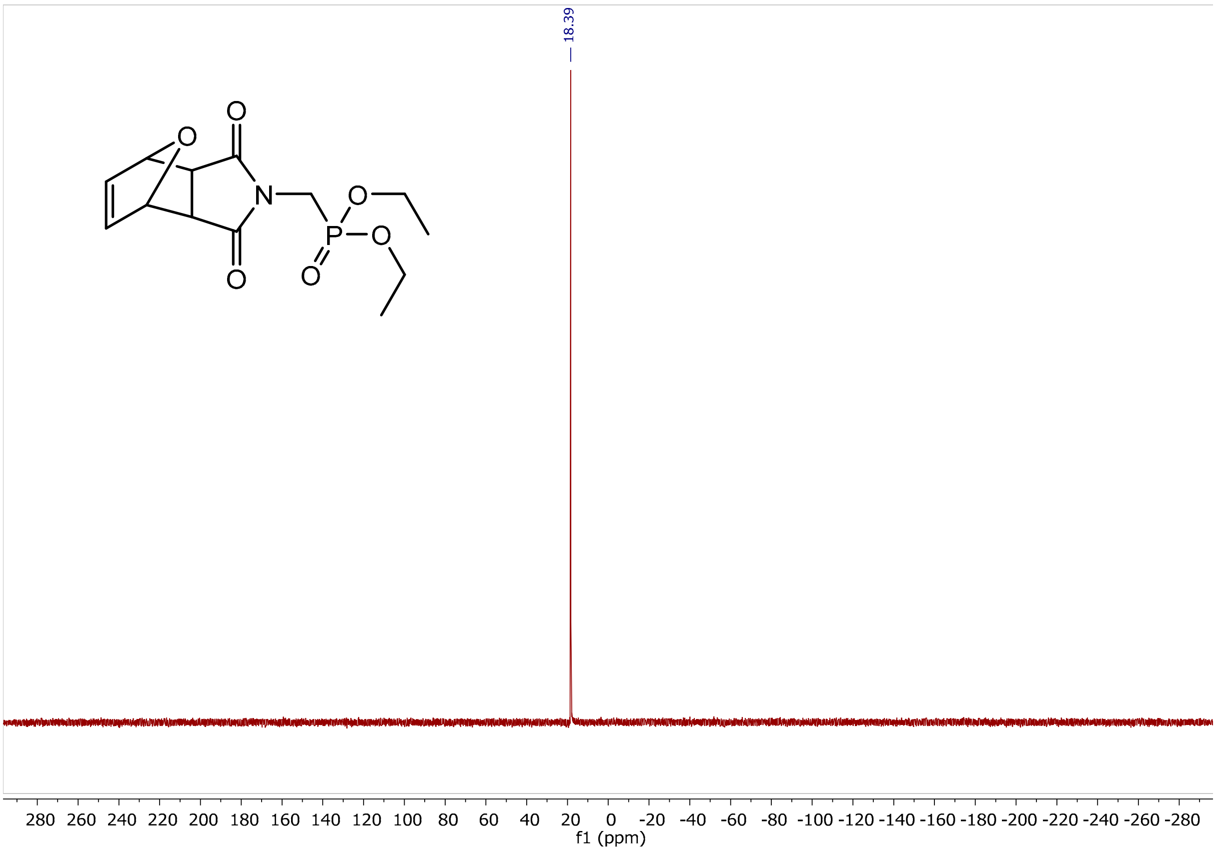

Figure S2. ^{31}P NMR spectrum of phosphonate ester-based monomer (Monomer 1)

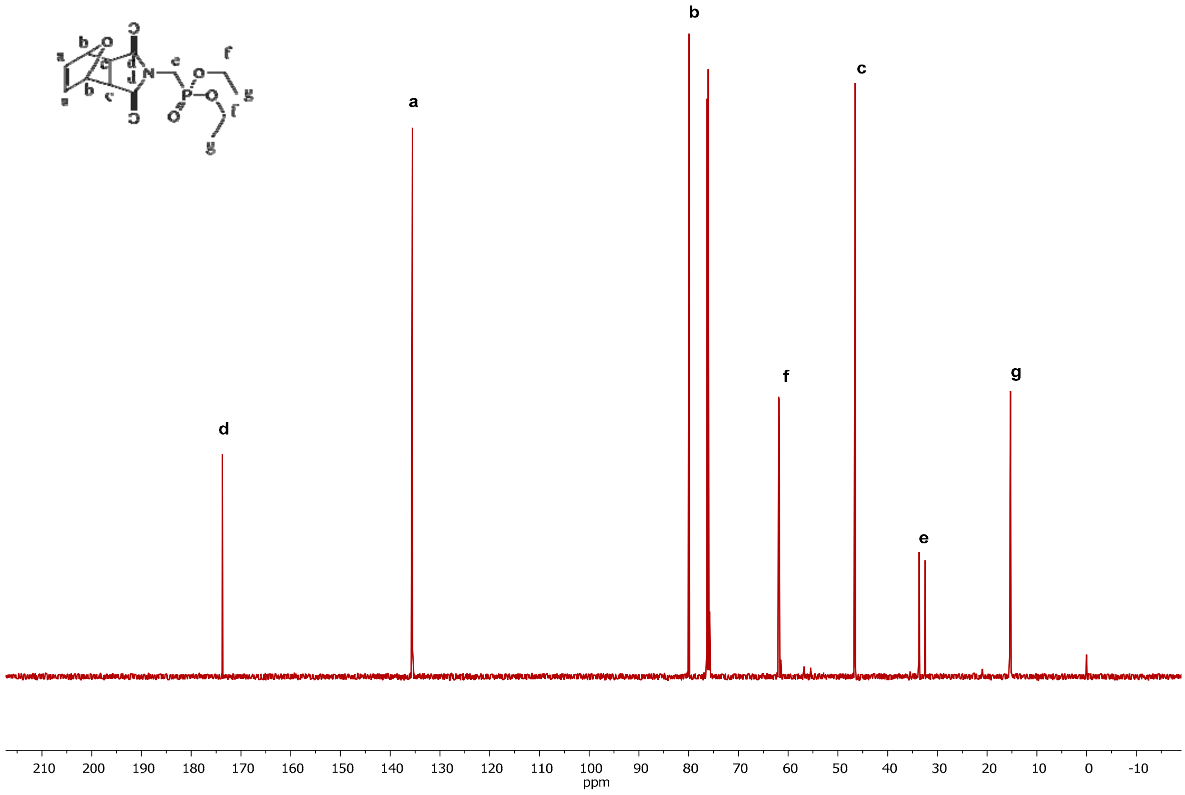

Figure S3. $^{13}C\{^{1}H\}$ NMR spectrum of phosphonate ester-based monomer (Monomer 1)

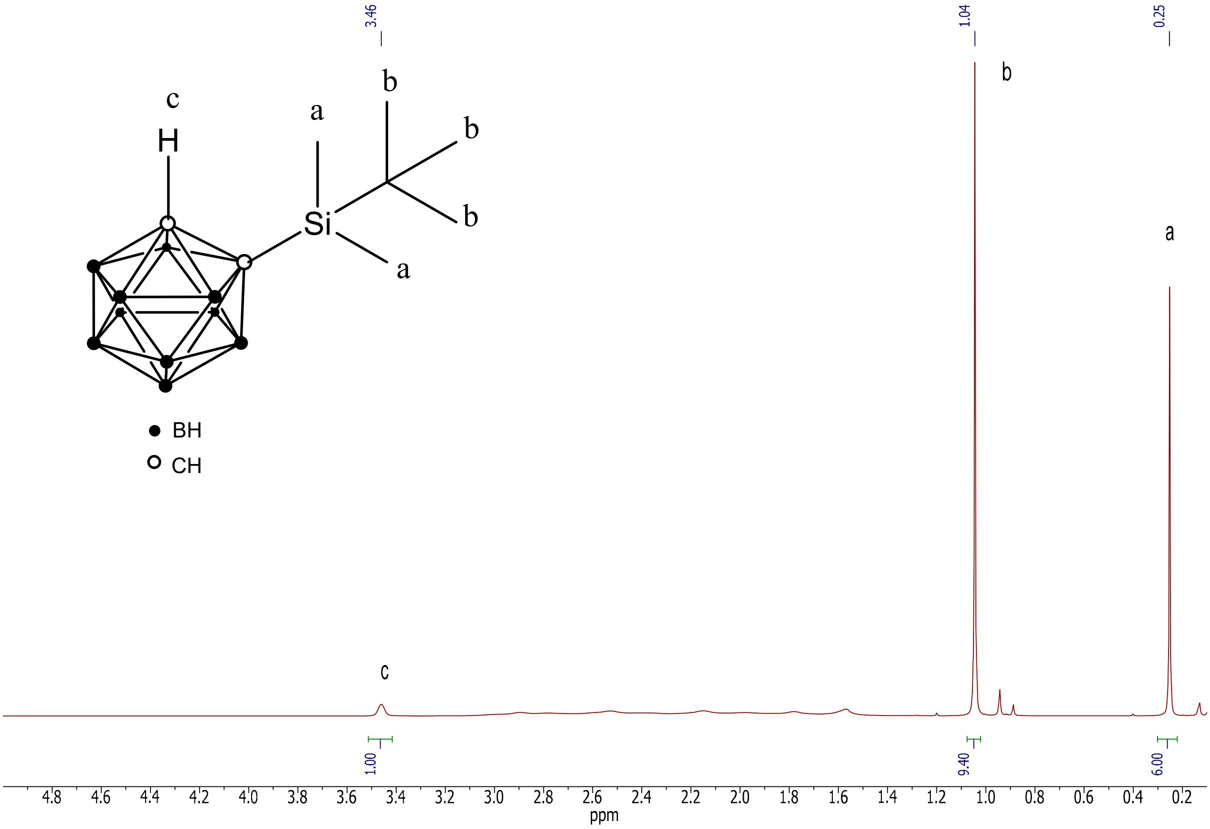

Figure S4. ^{1}H NMR spectrum of compound A

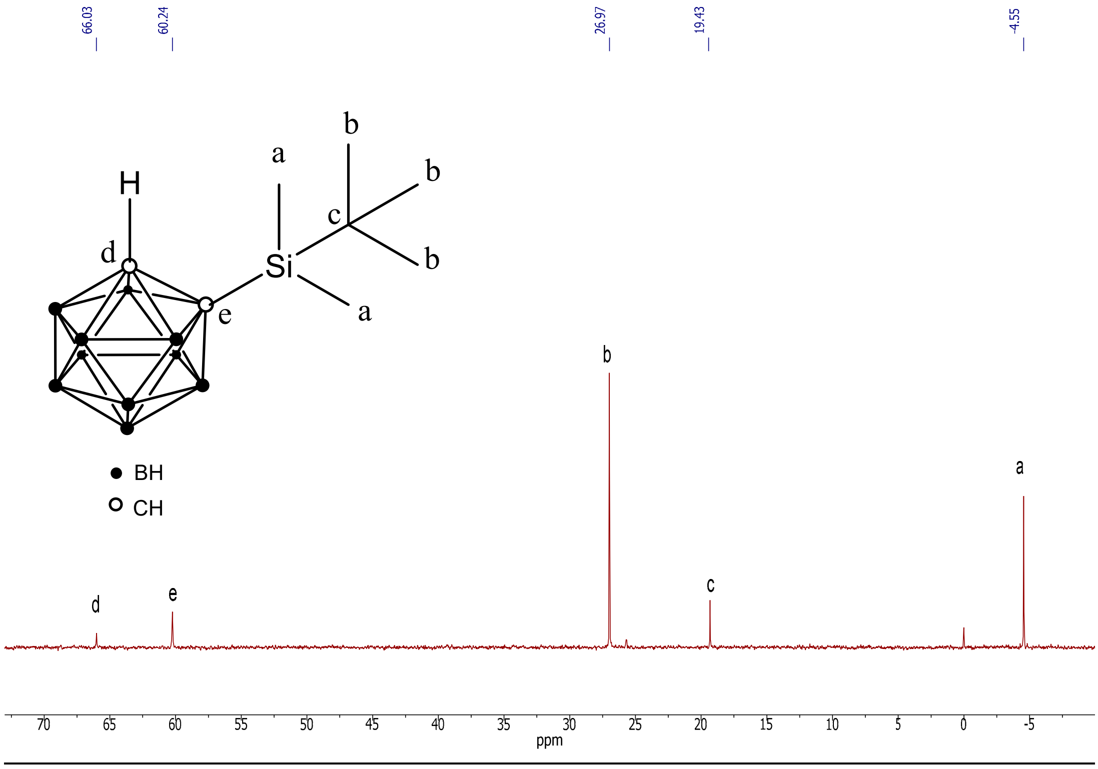

Figure S5. $^{13}C\{^{1}H\}$ NMR spectrum of compound A

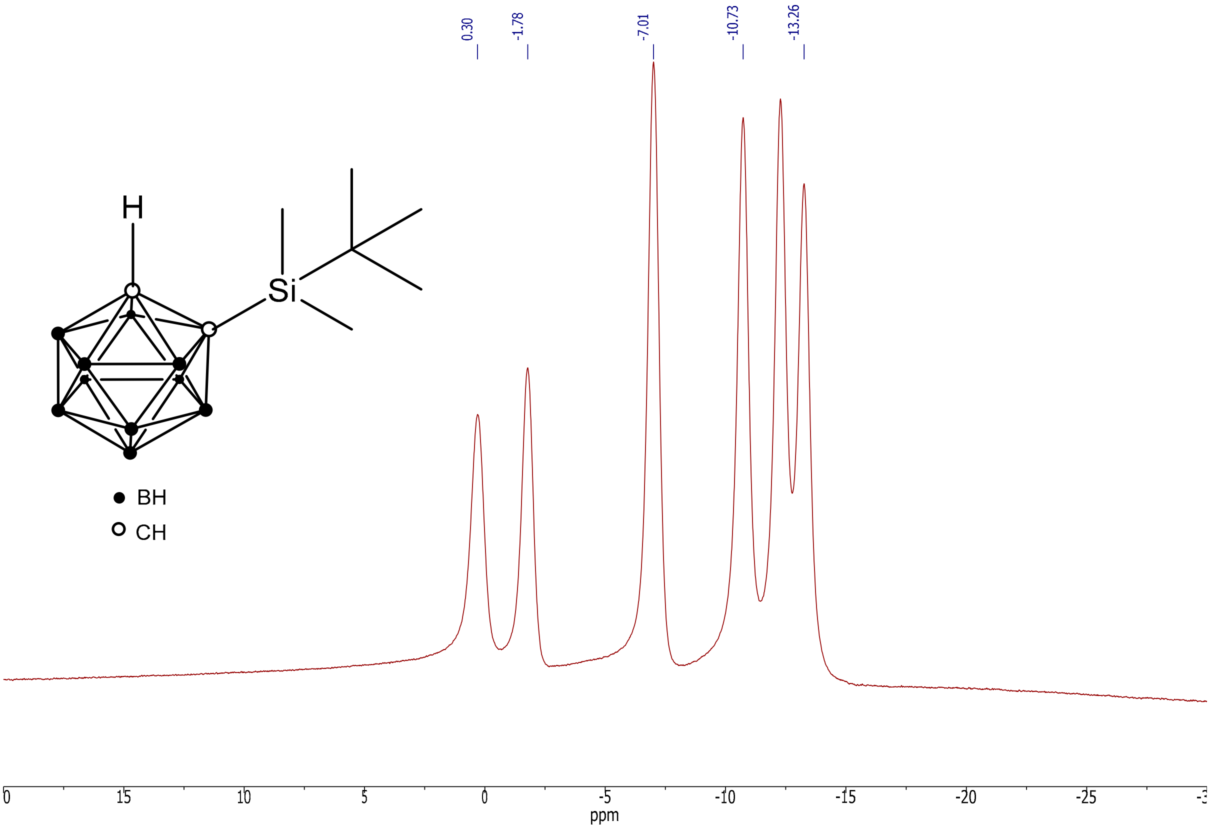

Figure S6. $^{11}B\{^{1}H\}$ NMR spectrum of compound A

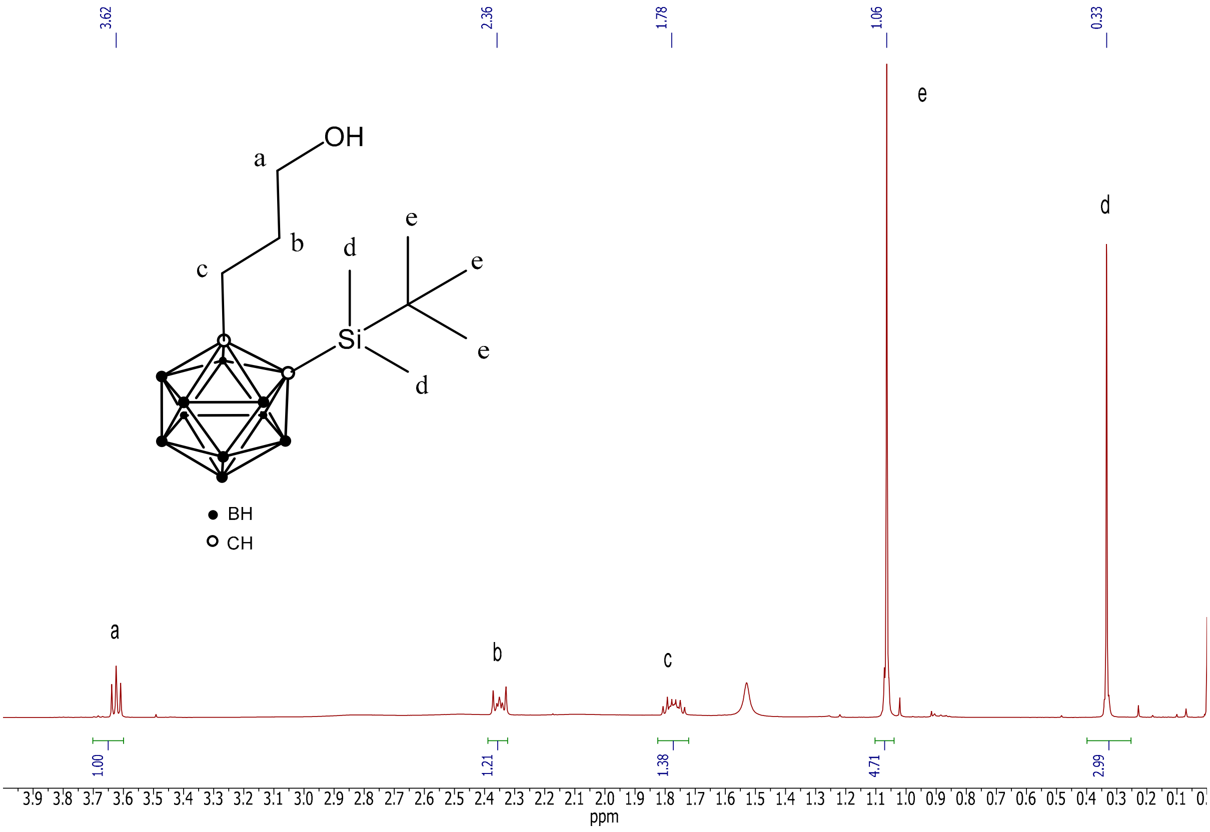

Figure S7. ^{1}H NMR spectrum of compound B

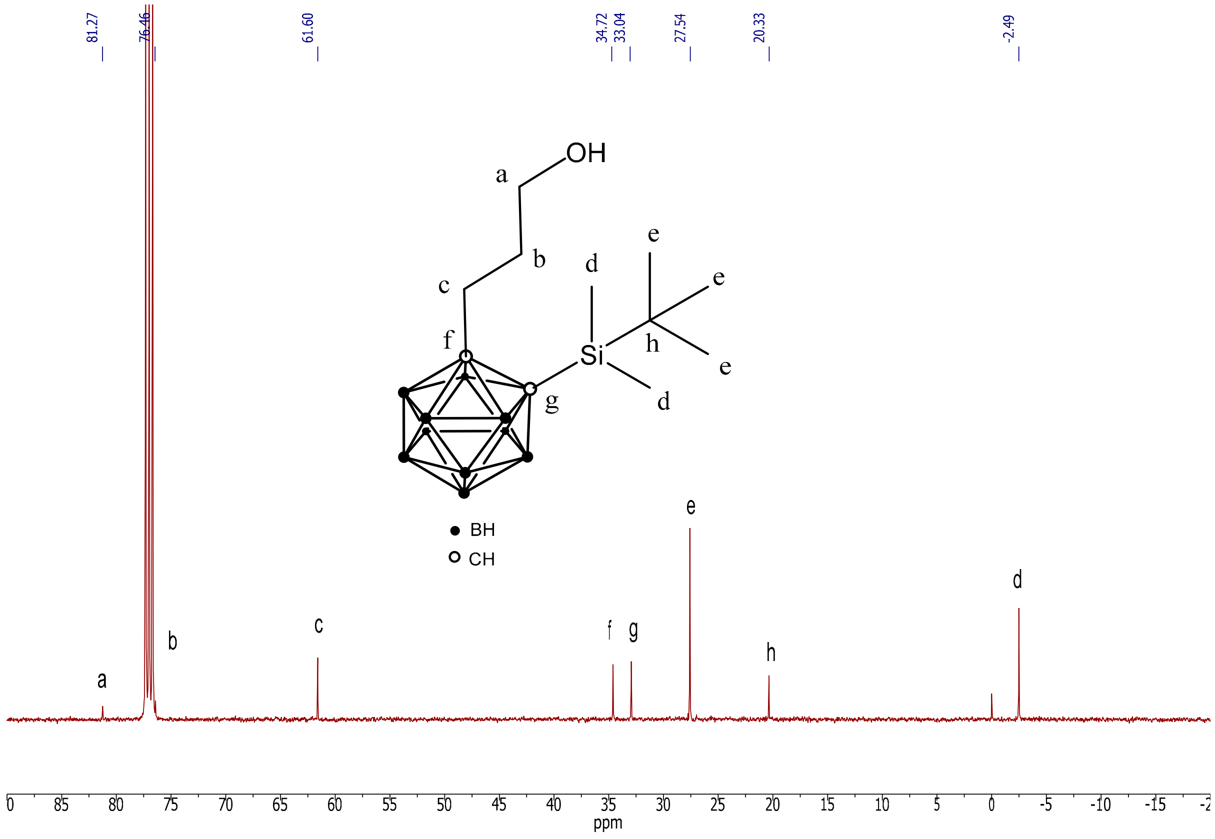

Figure S8. $^{13}C\{^{1}H\}$ NMR spectrum of compound B

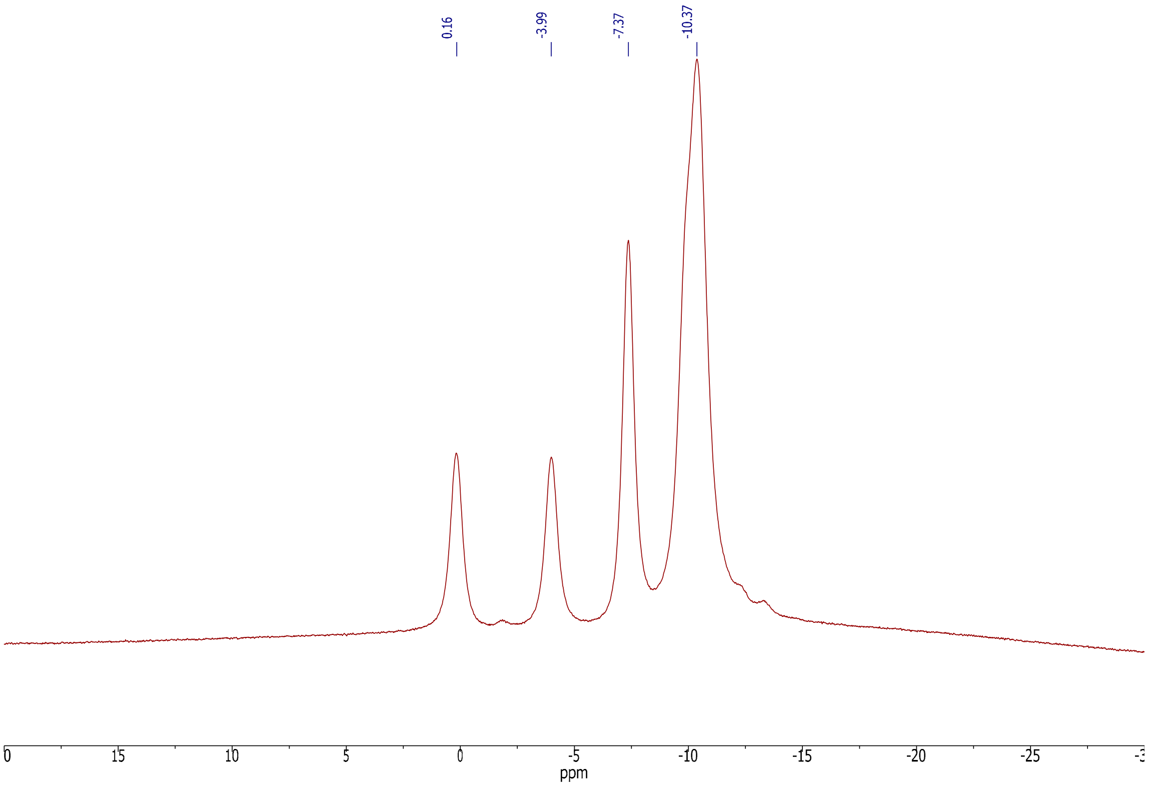

Figure S9. $^{11}B\{^{1}H\}$ NMR spectrum of compound B

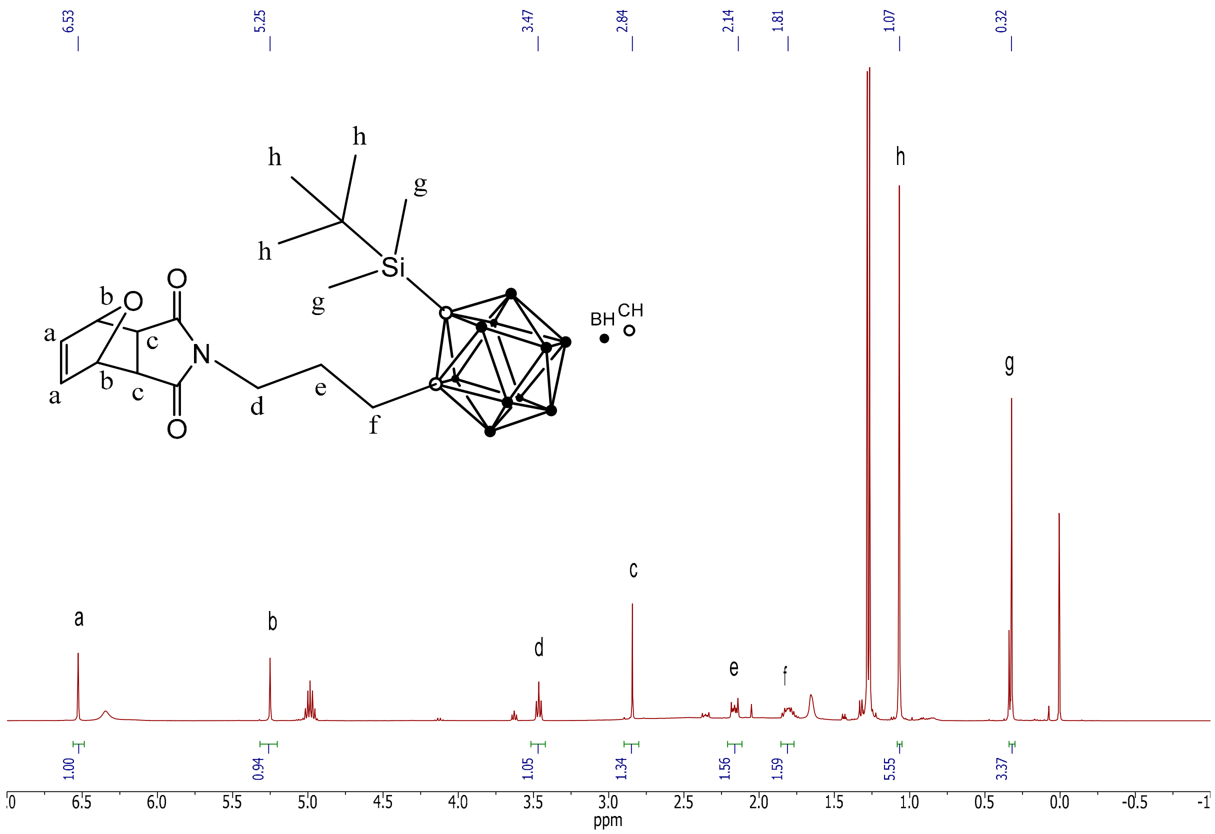

Figure S10. ^{1}H NMR spectrum of carbaborane-based monomer (Monomer 2)

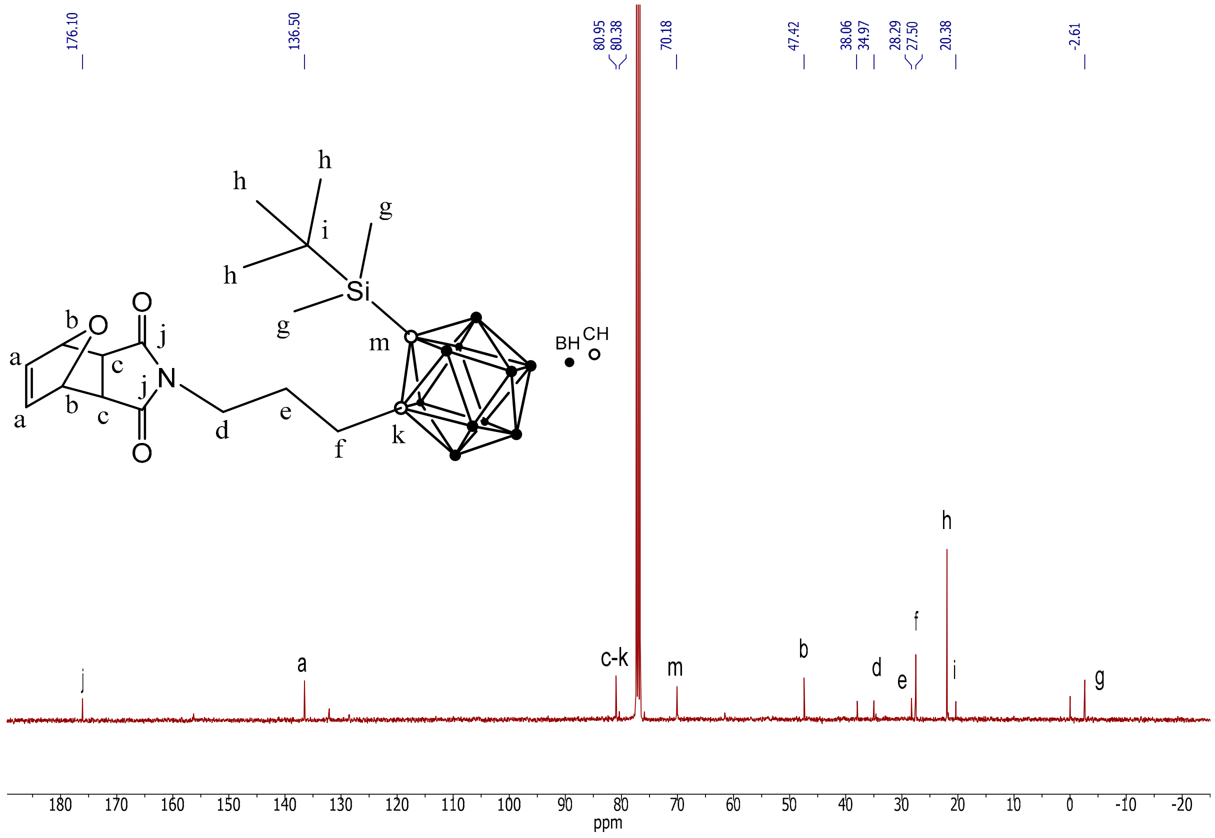

Figure S11. $^{13}C\{^{1}H\}$ NMR spectrum of carbaborane-based monomer (Monomer 2)

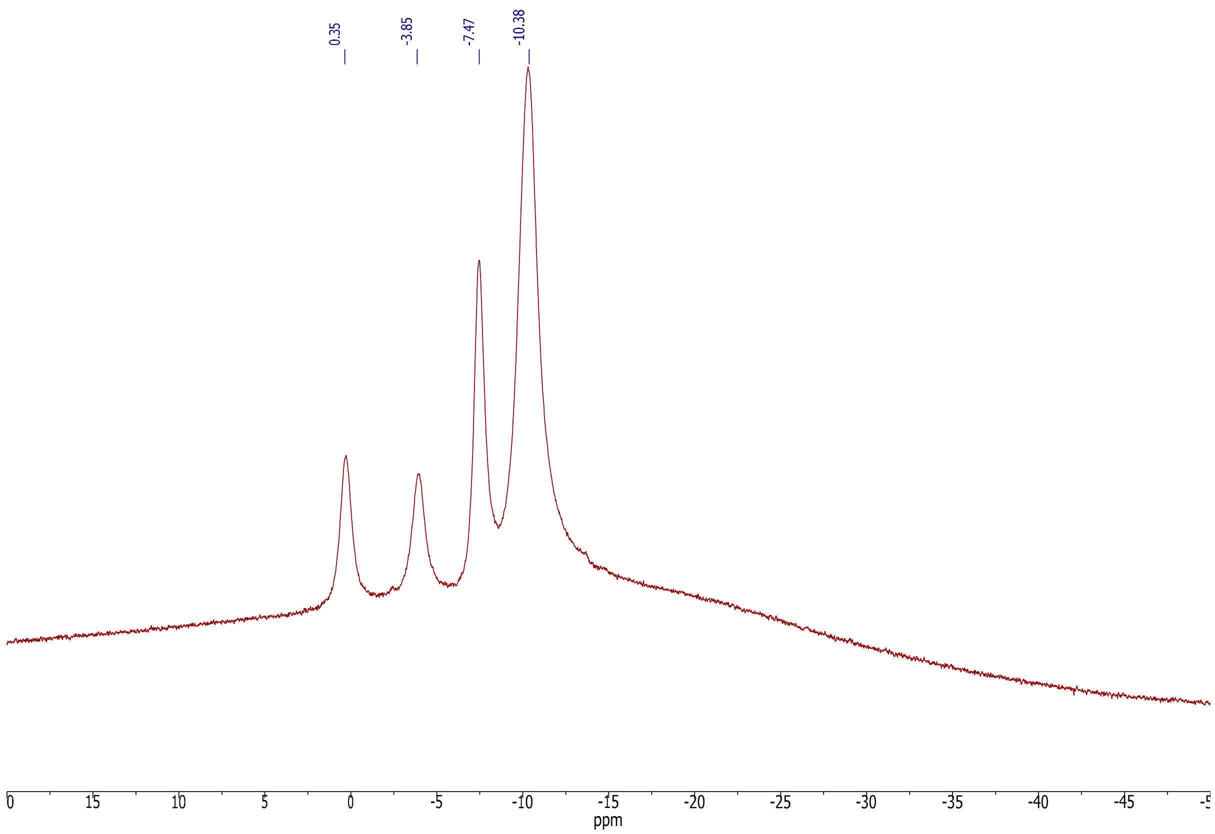

Figure S12. $^{11}B\{^{1}H\}$ NMR spectrum of carbaborane-based monomer (Monomer 2)

Polymers

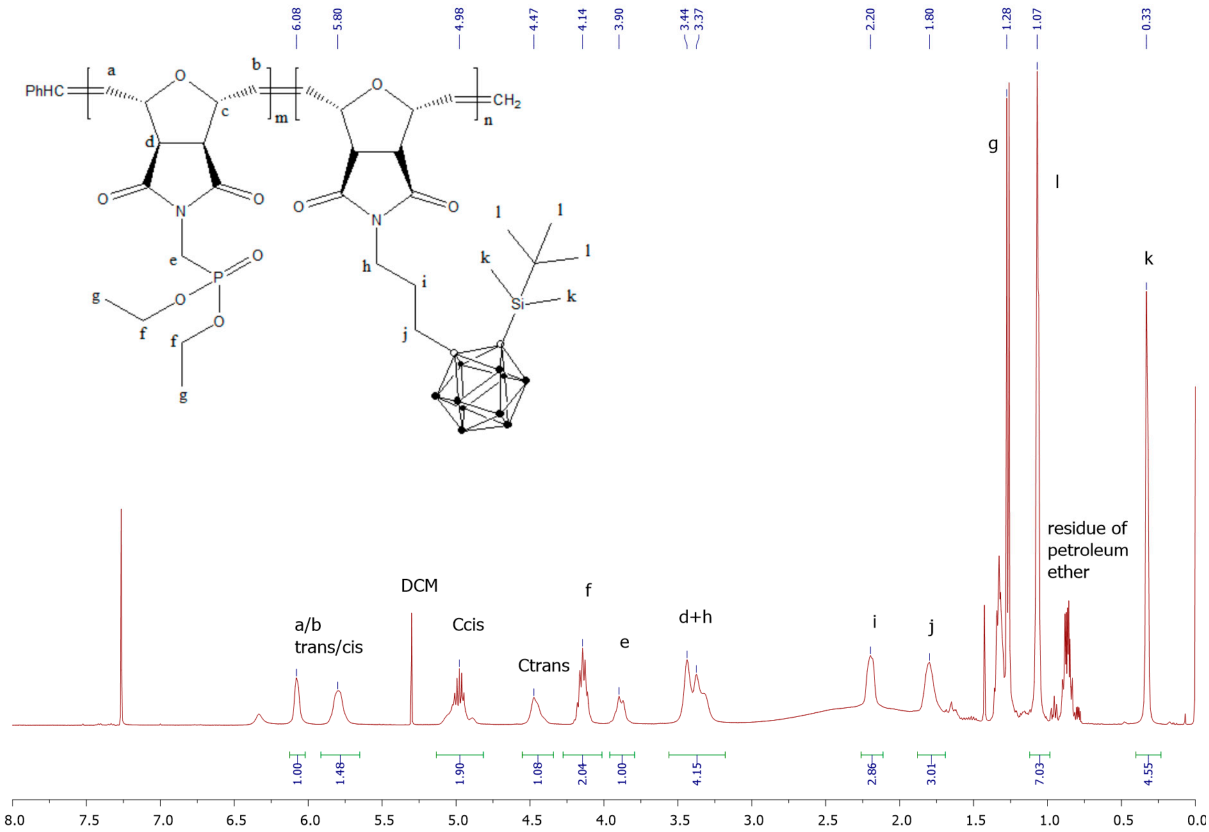

Figure S13. ^{1}H NMR spectrum of phosphonate ester- and carbaborane-based block copolymer with a theoretical ratio m:n (1:4) in $CDCl_3$

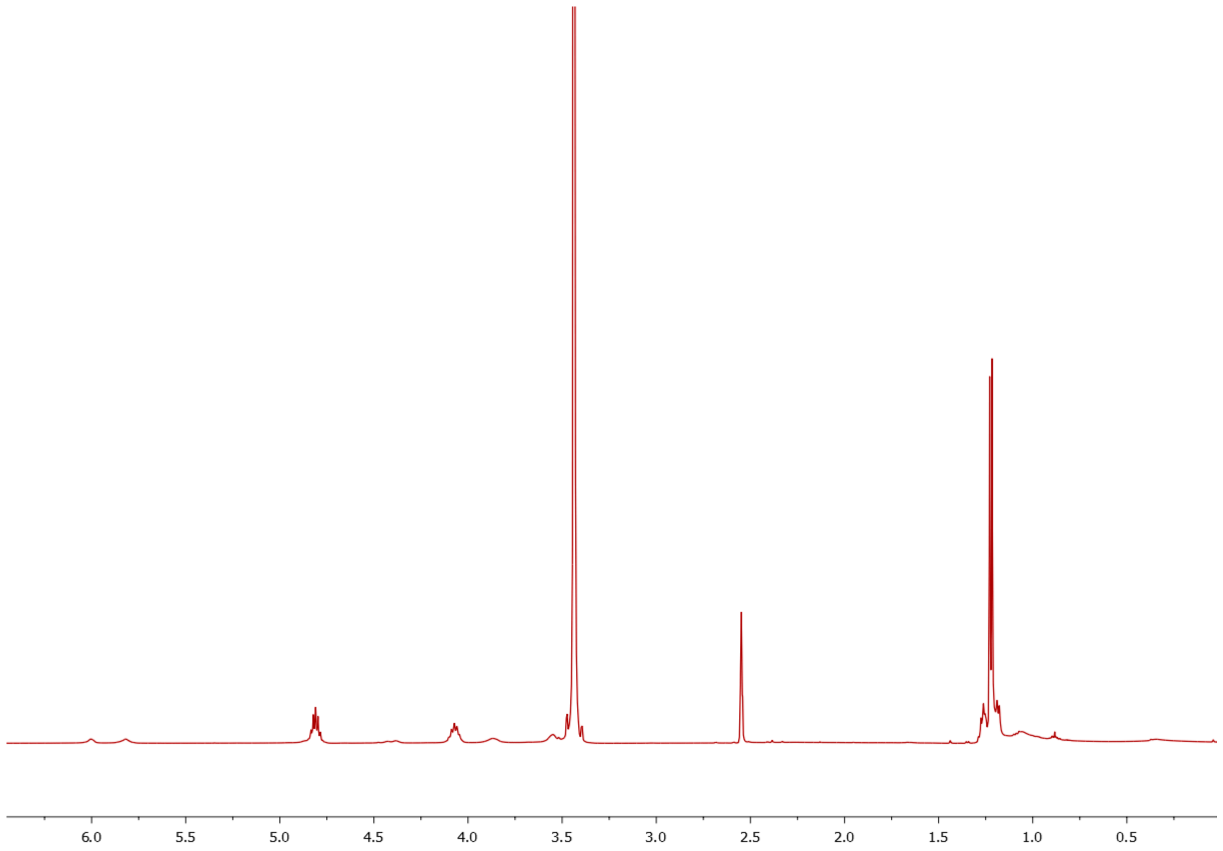

Figure S14. ^{1}H NMR of phosphonate ester- and carbaborane-based block copolymer with a theoretical ratio m:n (1:4) in DMSO-d_6

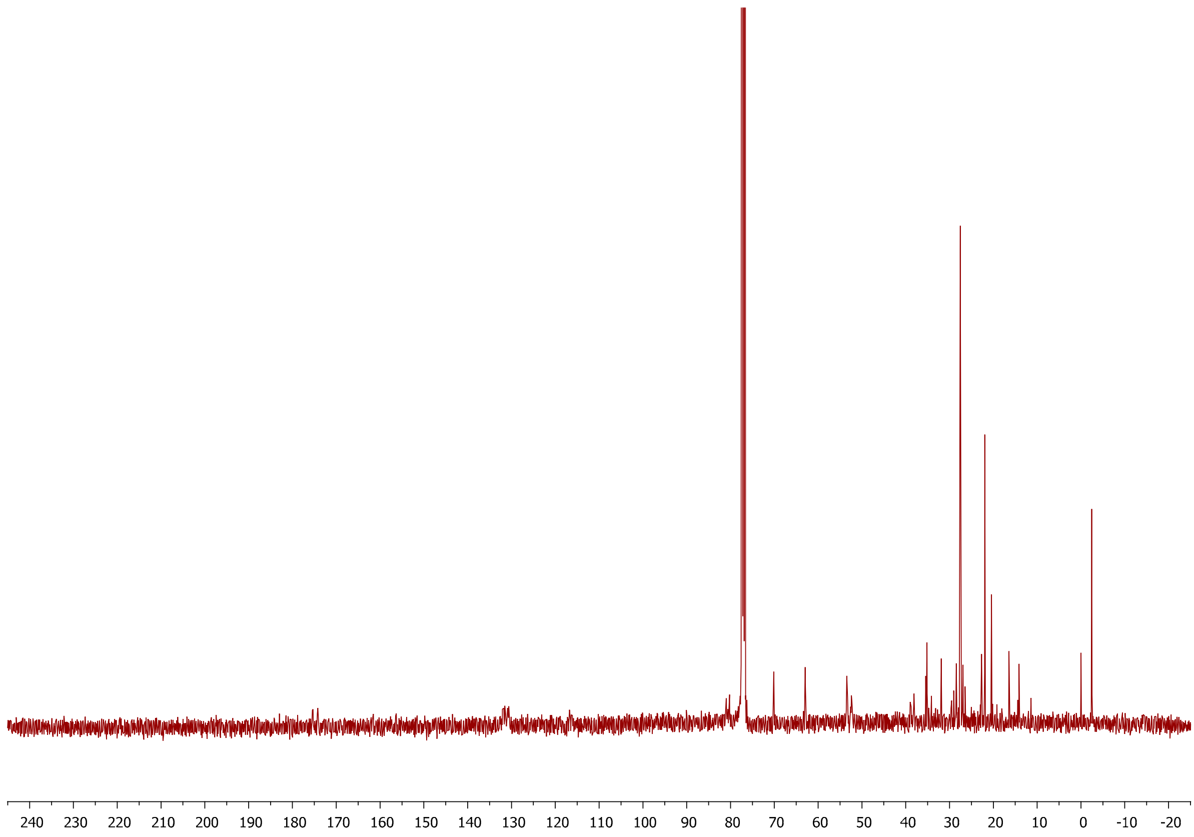

Figure S15. $^{13}C\{^{1}H\}$ NMR spectrum of phosphonate ester and carbaborane-based block copolymer with a theoretical ratio m:n (1:4)

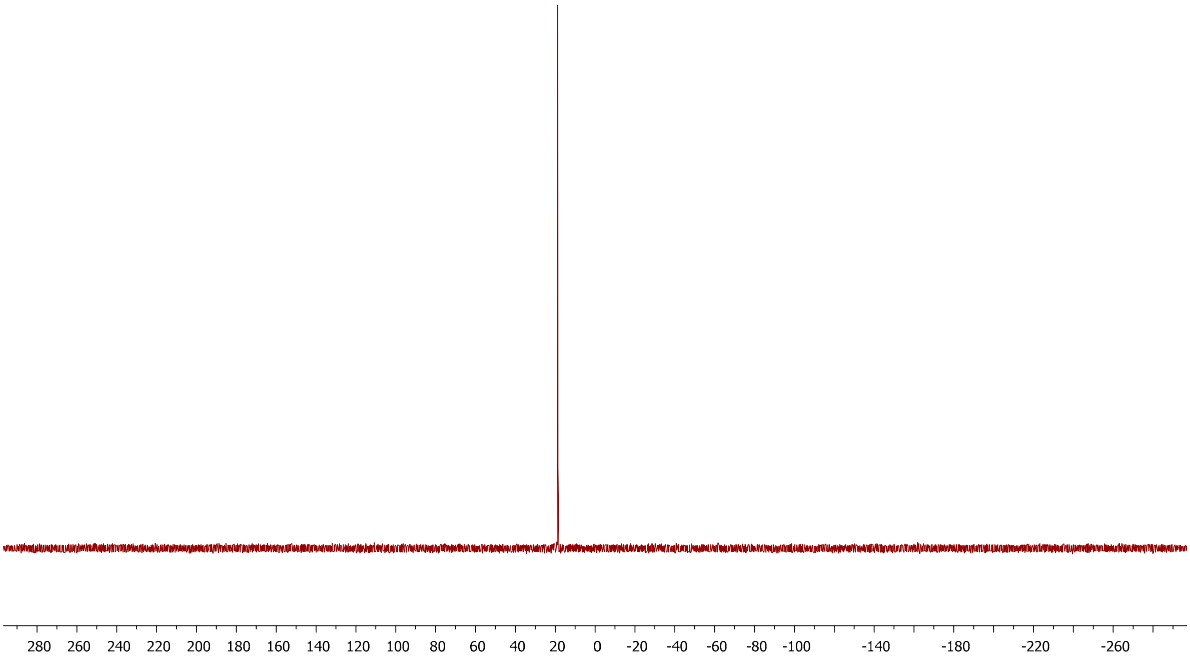

Figure S16. $^{31}P\{^{1}H\}$ NMR spectrum of phosphonate ester- and carbaborane-vbased block copolymer with a theoretical ratio m:n (1:4)

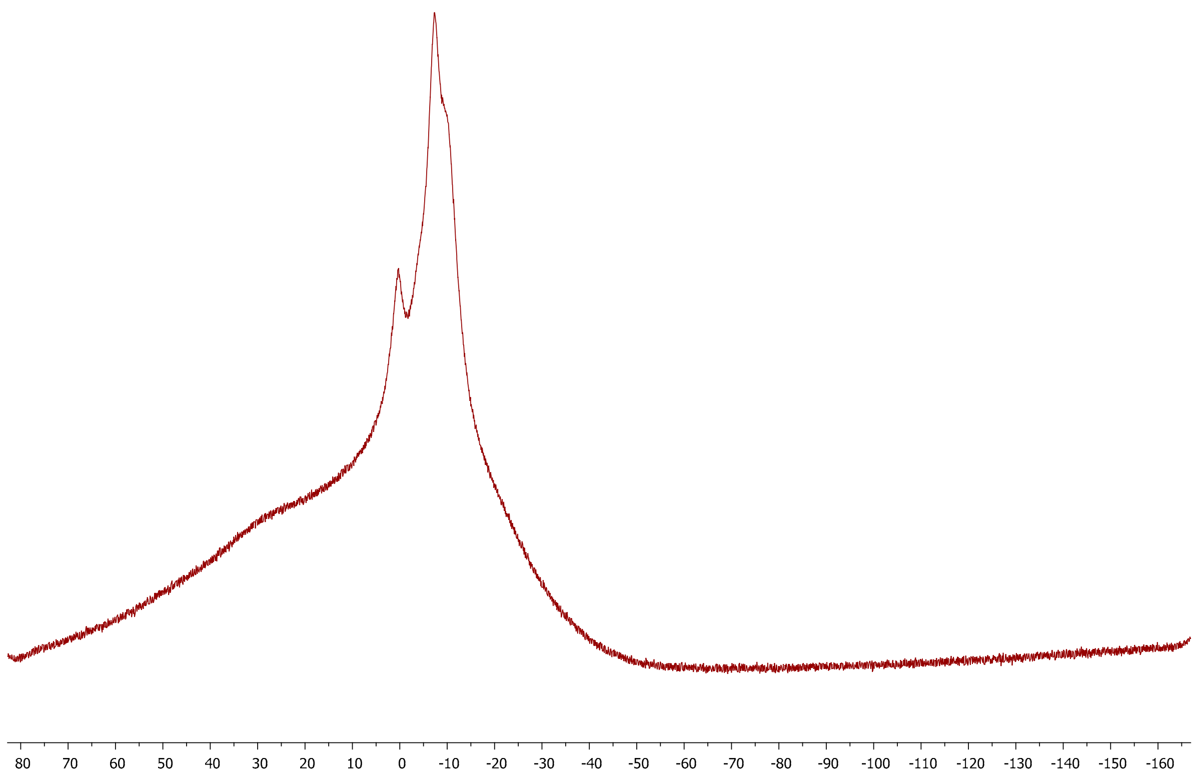

Figure S17. $^{11}B\{^{1}H\}$ NMR spectrum of phosphonate ester- and carbaborane-based block copolymer with a theoretical ratio m:n (1:4)

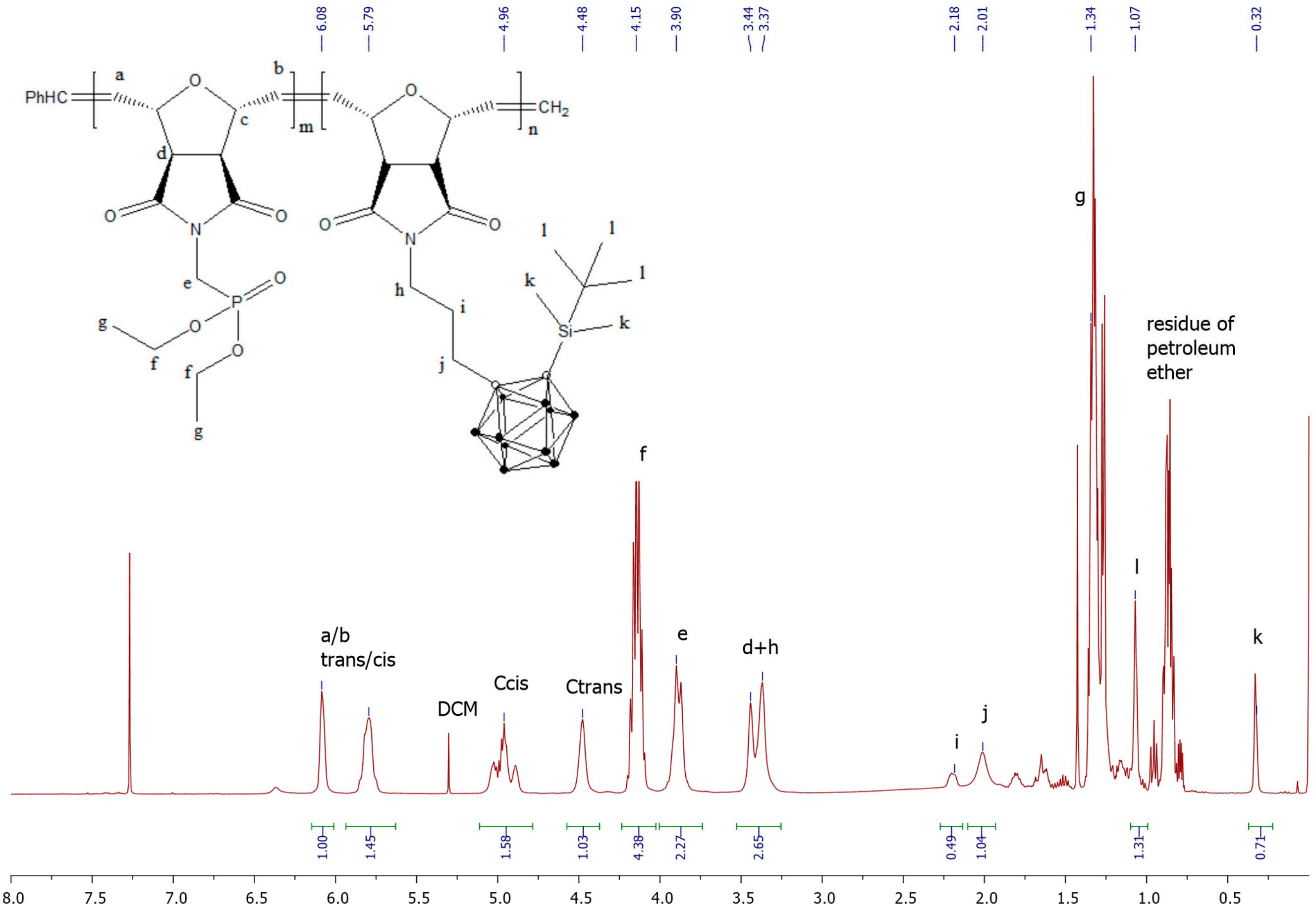

Figure S18. ^{1}H NMR spectrum of phosphonate ester- and carbaborane-based block copolymer with a theoretical ratio m:n (4:1)

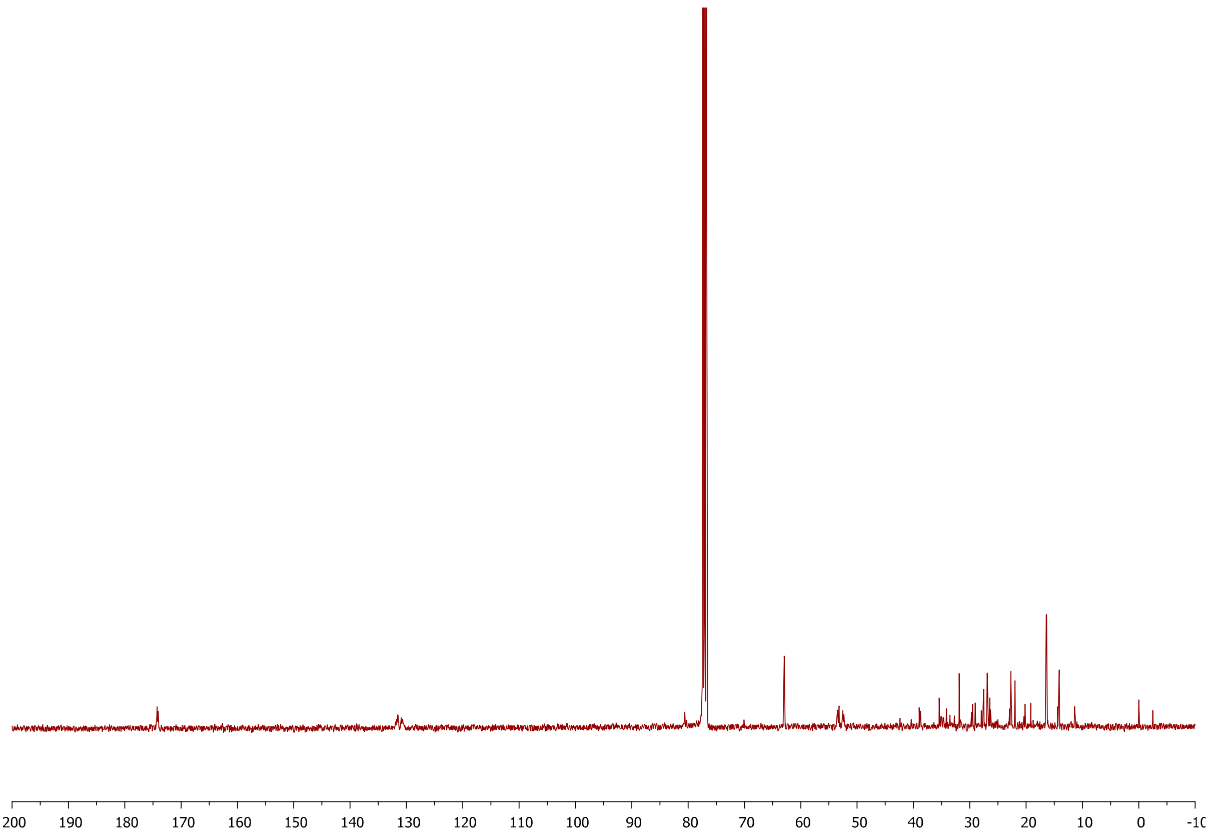

Figure S19. $^{13}C\{^{1}H\}$ NMR spectrum of phosphonate ester- and carbaborane-based block copolymer with a theoretical ratio m:n (4:1)

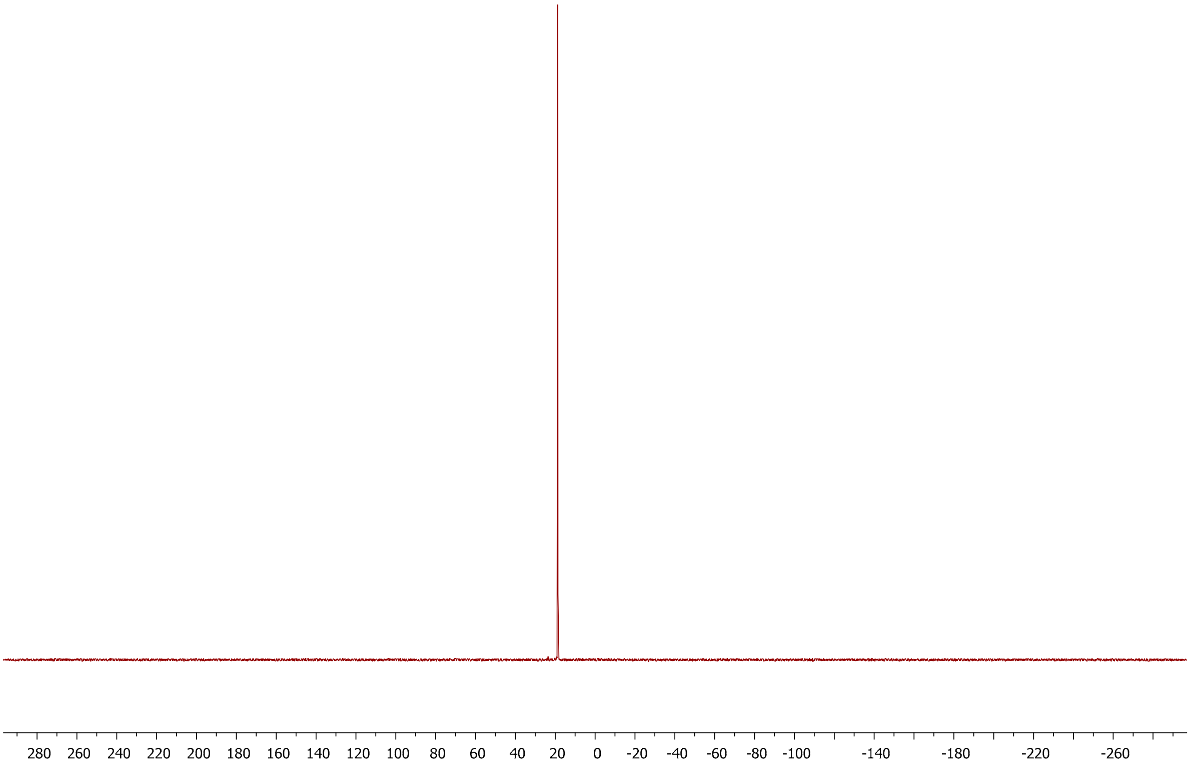

Figure S20. $^{31}P\{^{1}H\}$ NMR spectrum of phosphonate ester- and carbaborane-based block copolymer with a theoretical ratio m:n (4:1)

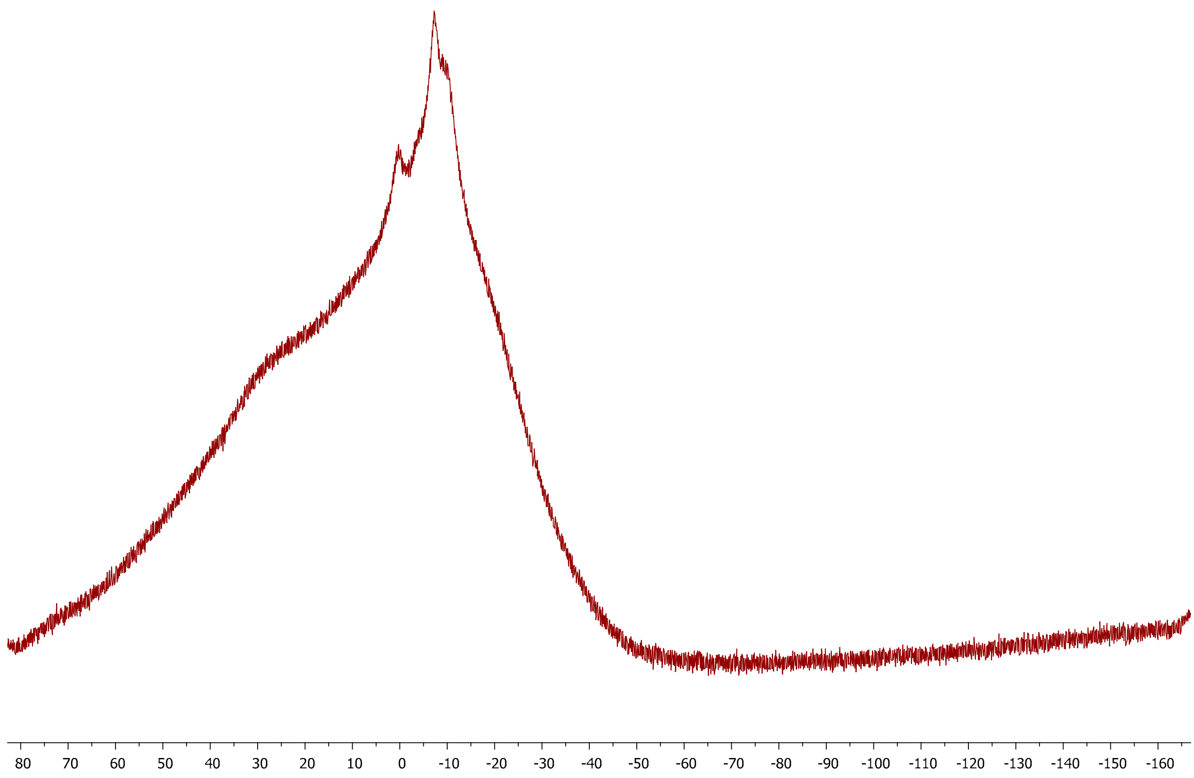

Figure S21. $^{11}B\{^{1}H\}$ NMR spectrum of phosphonate ester- and carbaborane-based block copolymer with a theoretical ratio m:n (4:1)

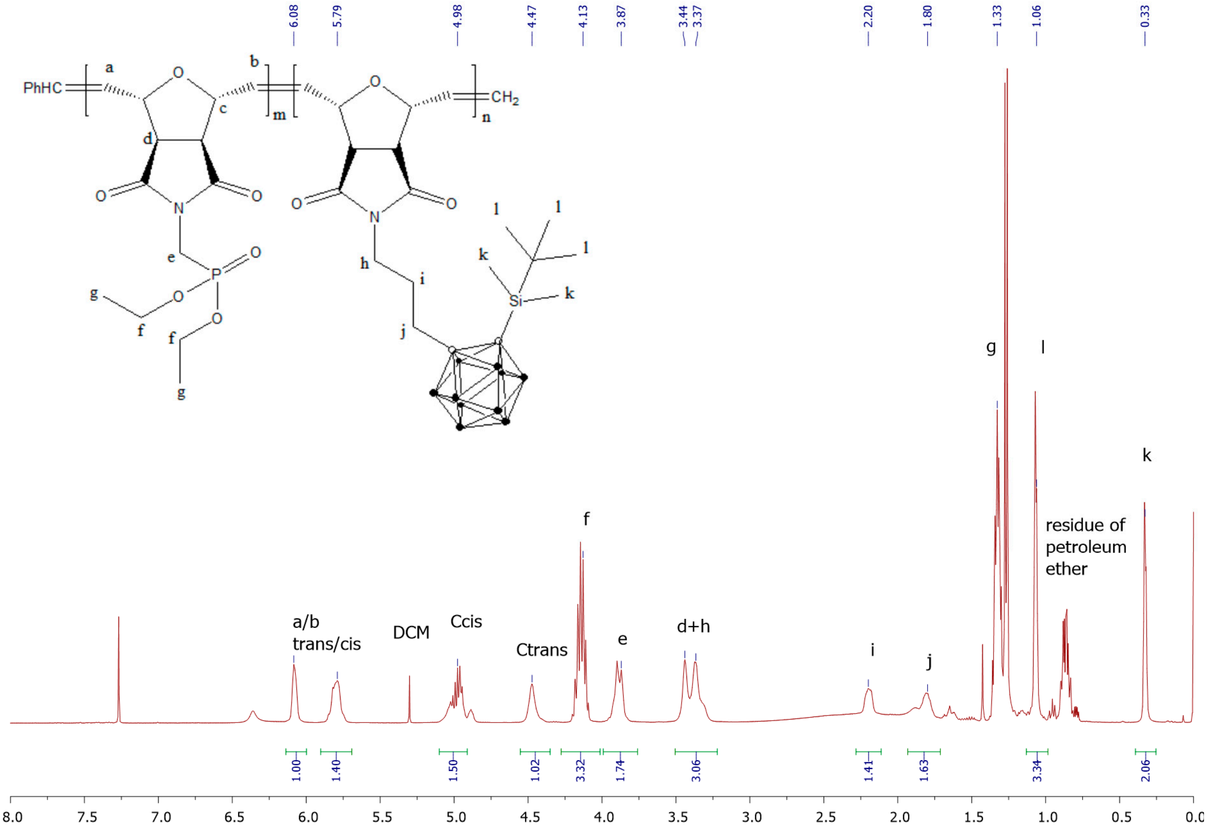

Figure S22. ^{1}H NMR spectrum of phosphonate ester- and carbaborane-based block copolymer with a theoretical ratio m:n (1:1)

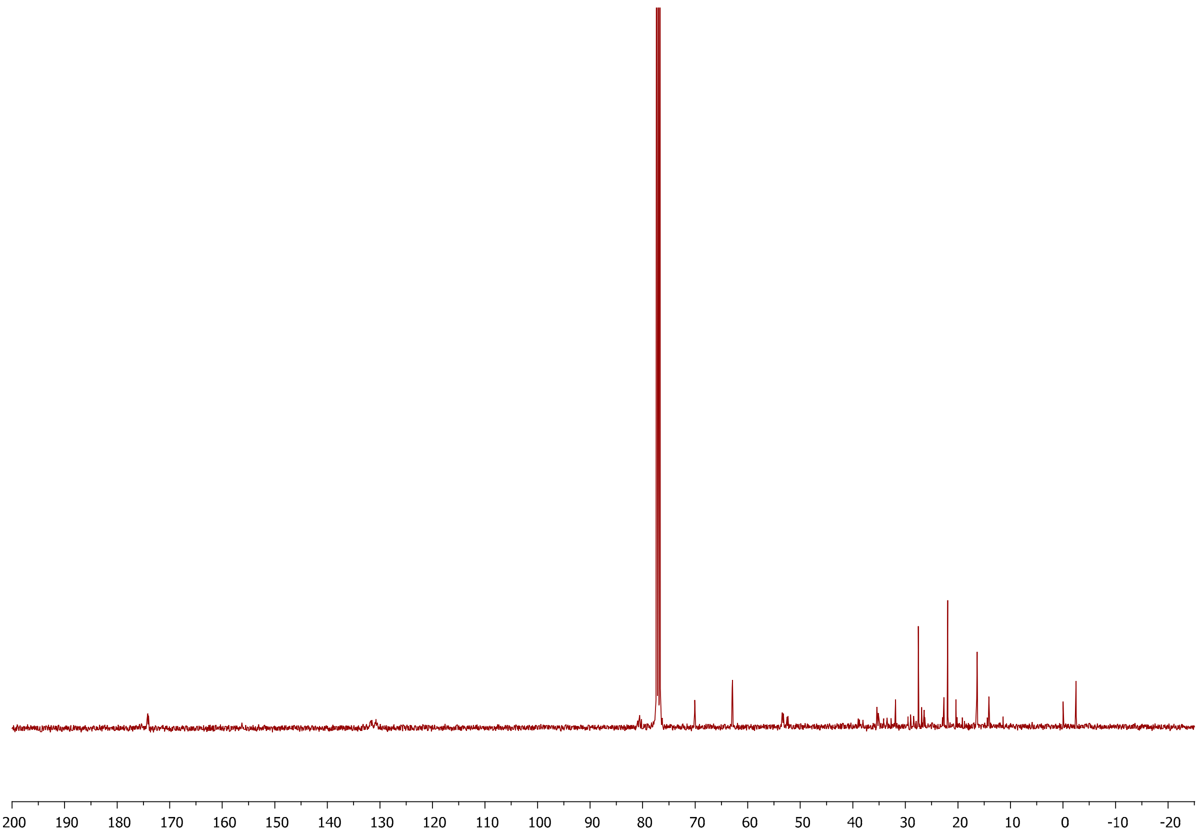

Figure S23. $^{13}C\{^{1}H\}$ NMR spectrum of phosphonate ester- and carbaborane-based block copolymer with a theoretical ratio m:n (1:1)

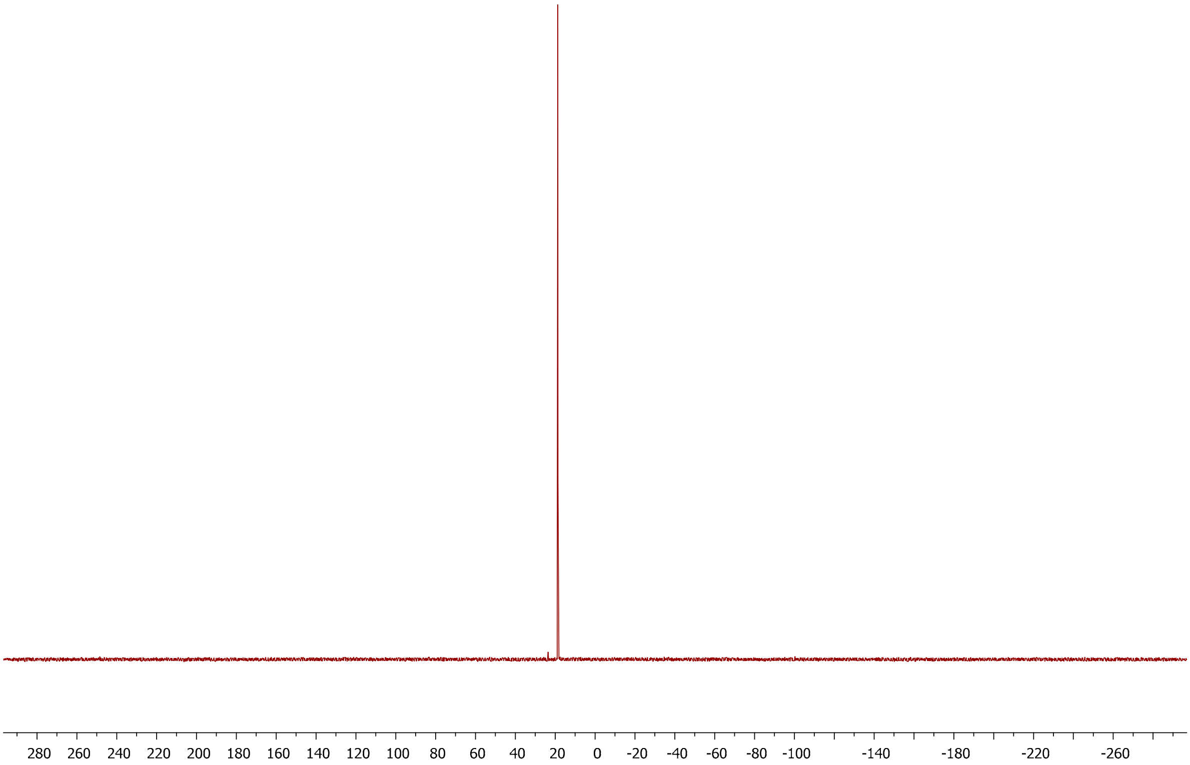

Figure S24. $^{31}P\{^{1}H\}$ NMR spectrum of phosphonate ester- and carbaborane-based block copolymer with a theoretical ratio m:n (1:1)

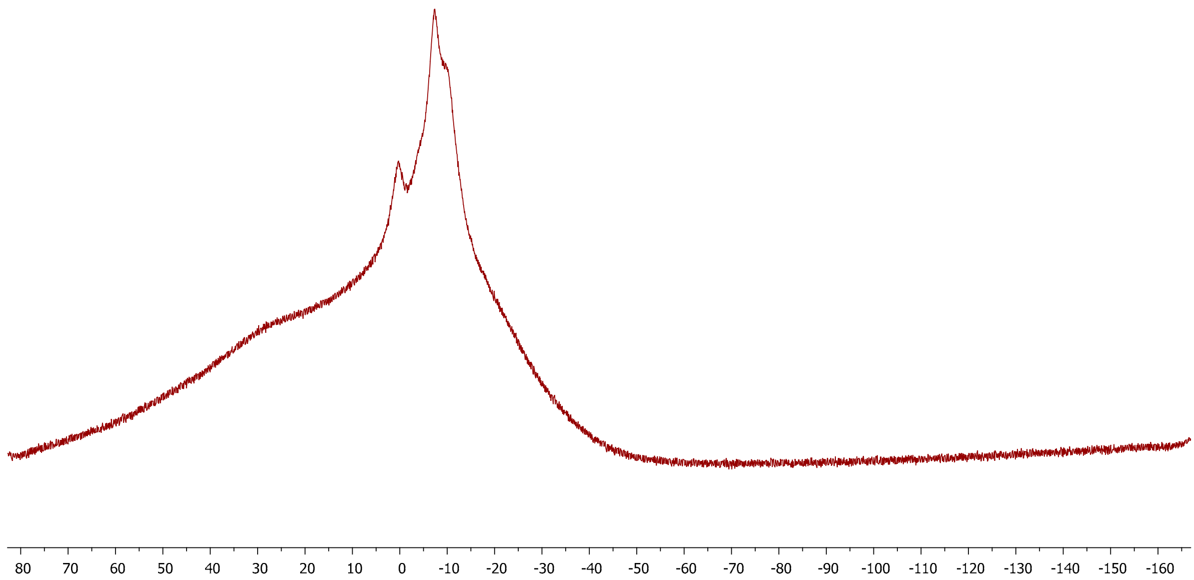

Figure S25. $^{11}B\{^{1}H\}$ NMR spectrum of phosphonate ester- and carbaborane-based block copolymer with a theoretical ratio m:n (1:1)

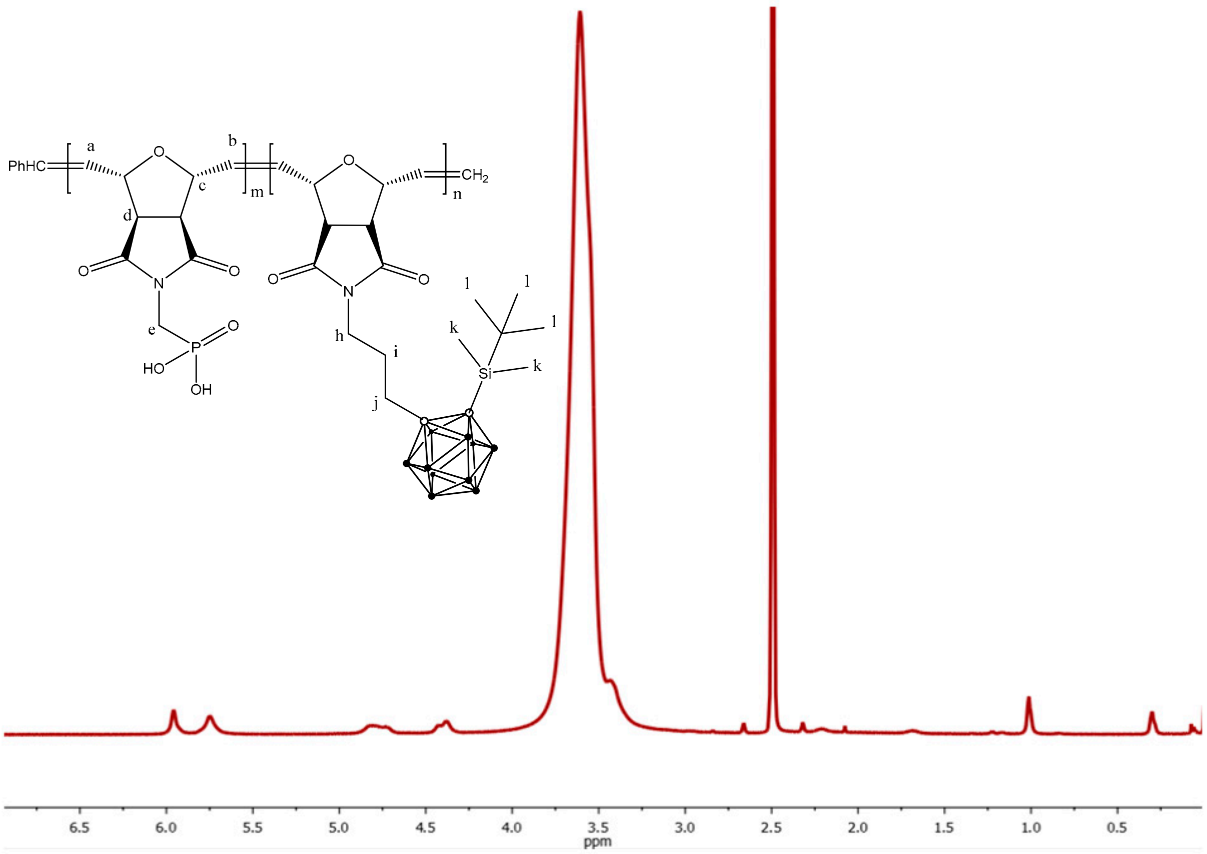

Figure S26. ^{1}H NMR spectrum of phosphonic acid- and carbaborane-based block copolymer with a theoretical ratio m:n (4:1)

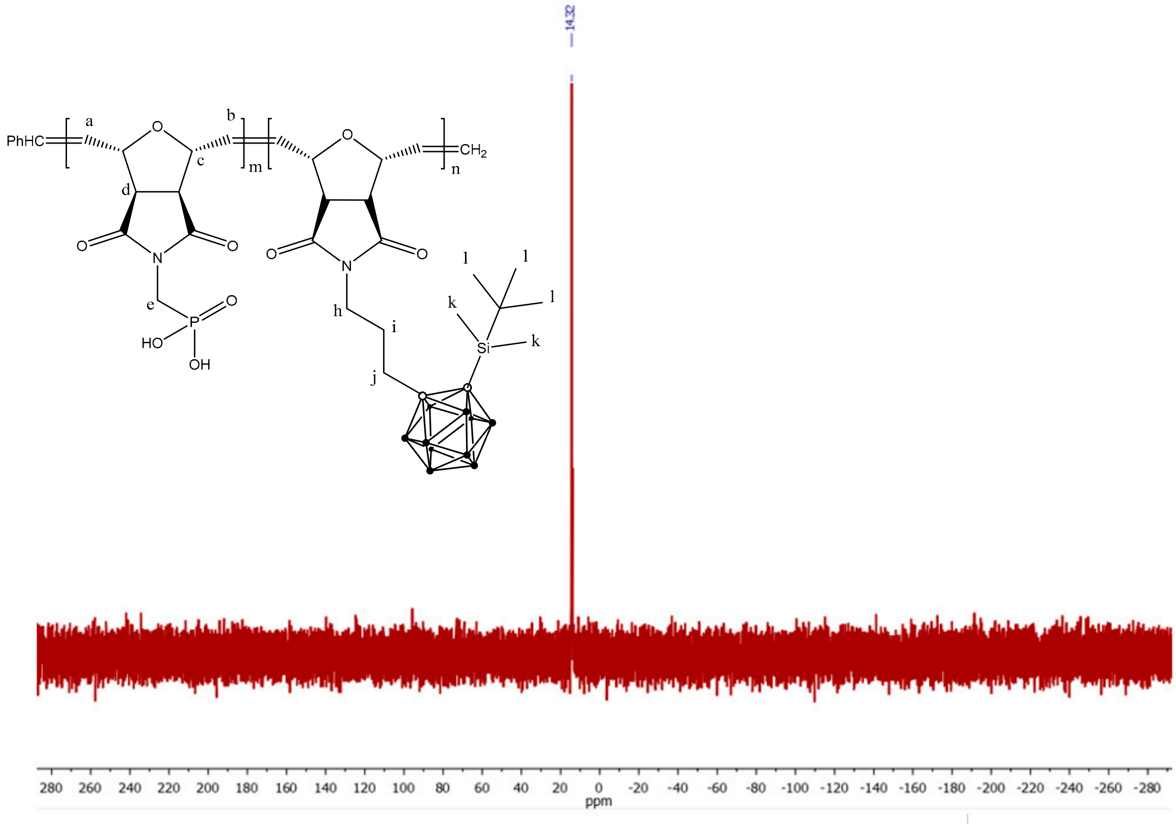

Figure S27. $^{31}P\{^{1}H\}$ NMR spectrum of phosphonic acid- and carbaborane-based block copolymer with a theoretical ratio m:n (4:1)

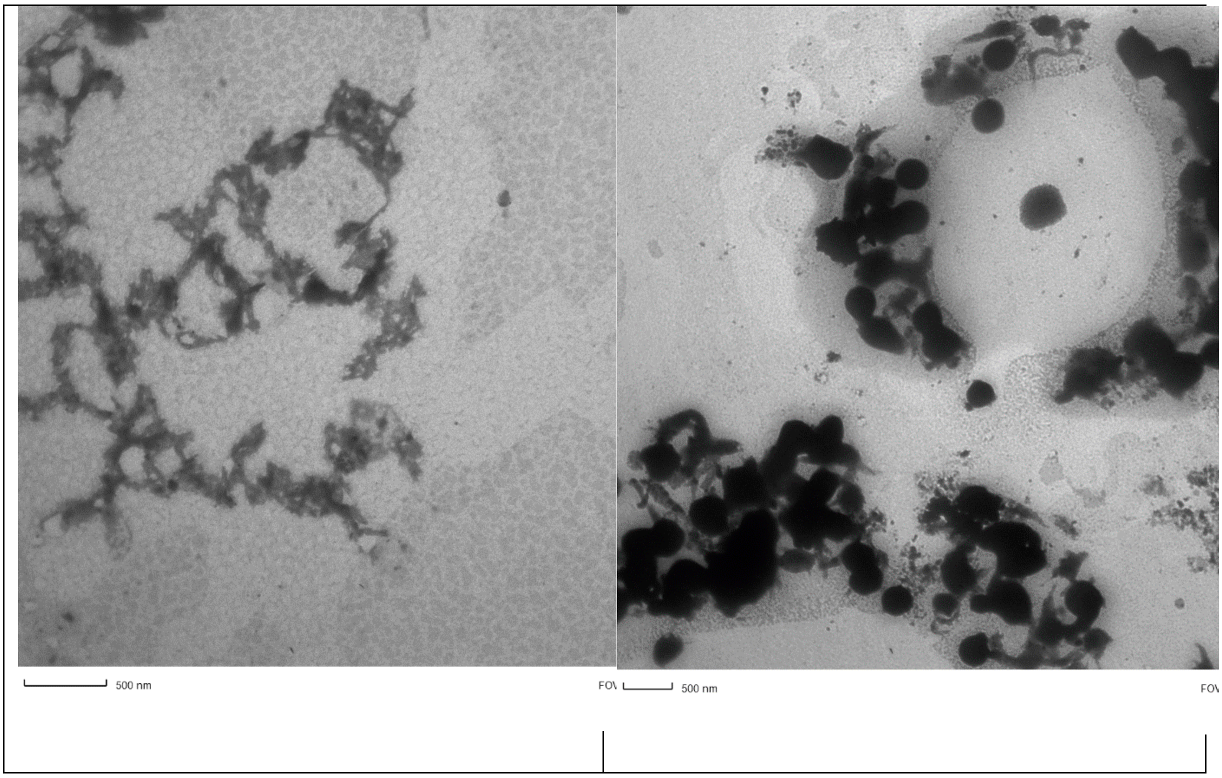

Figure S28. TEM observation of iron oxide nanoparticles coated with phosphonate-based polymer, **P2A**.

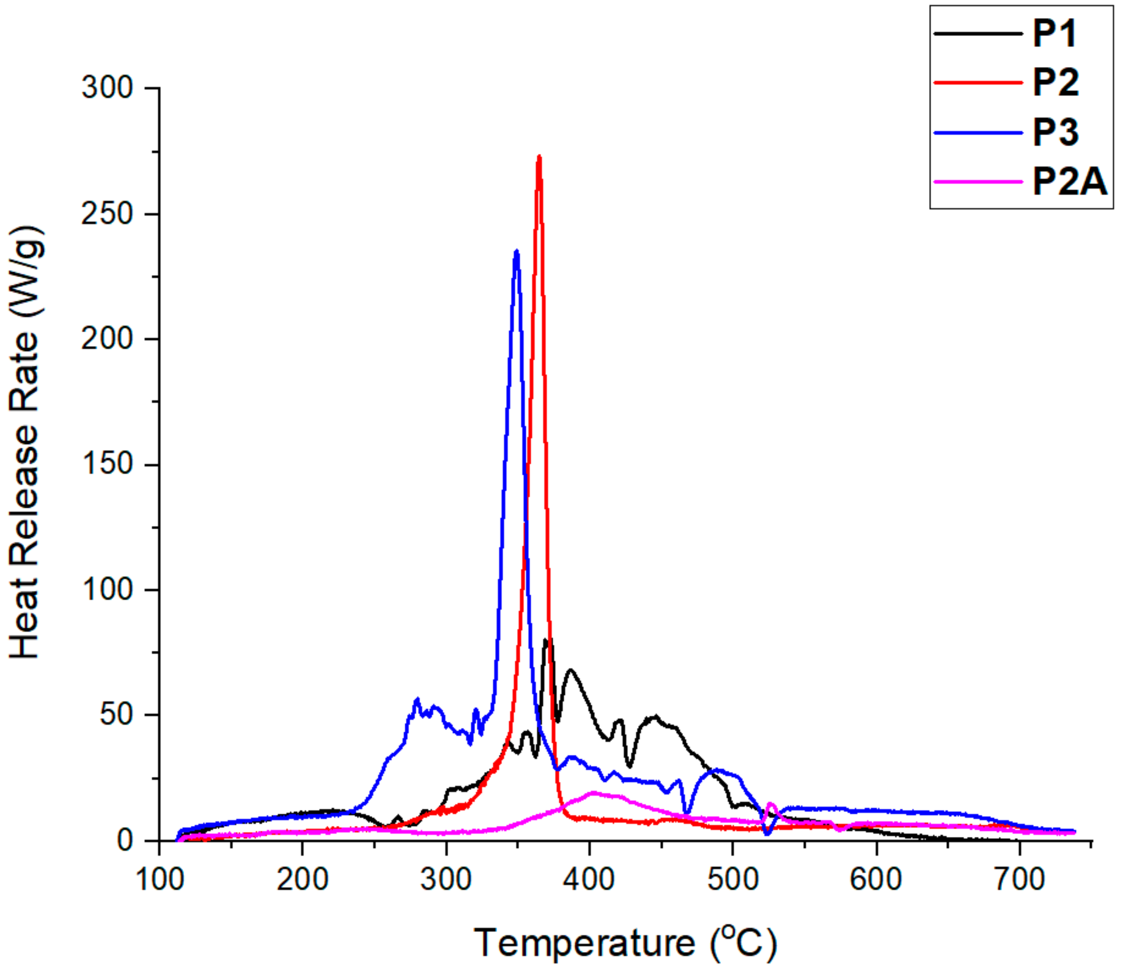

Figure S29. HRR curves of polymers from MCC testing

3. References

S1. Eren, T., Tew, G., J. Polym. Sci.: Part A: Polym. Chem. **2009**, 47, 3949-3956.

S2. Simon. Y, Ohm C., Zimny M. J., Coughlin E. B, Macromolecules **2007**, 40, 5628-5630.

S3. Love, J. A., Morgan, J. P., Trnka, T. M., R. H. Grubbs, Angew. Chem. 2002, 41, 4035-4037.
